# Dicationic ionic liquids as new feeding deterrents

**DOI:** 10.1007/s11696-018-0495-6

**Published:** 2018-05-16

**Authors:** Damian K. Kaczmarek, Kamil Czerniak, Tomasz Klejdysz

**Affiliations:** 1 Department of Chemical Technology, Poznan University of Technology, 60-965 Poznan, Poland; 2 Institute of Plant Protection - National Research Institute, 60-318 Poznan, Poland

**Keywords:** Quaternary bis(ammonium) salts, Dicationic ionic liquids, Antifeedant, Feeding deterrents

## Abstract

**Abstract:**

In this study, new quaternary bis(ammonium) salts with alkyl-1,X-bis(dimethyldecylammonium) cation and saccharinate, acesulfamate, lactate and pyroglutamate anions were synthesized and characterized by ^1^H and ^13^C NMR spectroscopy. Thermal gravimetric and differential scanning calorimetry analyses confirmed that all salts were thermally stable and the majority of them exhibited melting points below 100 °C. The physicochemical properties (viscosity, density, refractive index values, and solubility) of the obtained salts were determined for three compounds with lactate anions. All the tested salts have suitable properties which, in practical application, will reduce the losses caused by the most important storage insects. Most of the synthesized ionic liquids had comparable or better deterrent activity than azadirachtin—an alkaloid known as the most active antifeedant.

**Graphical abstract:**

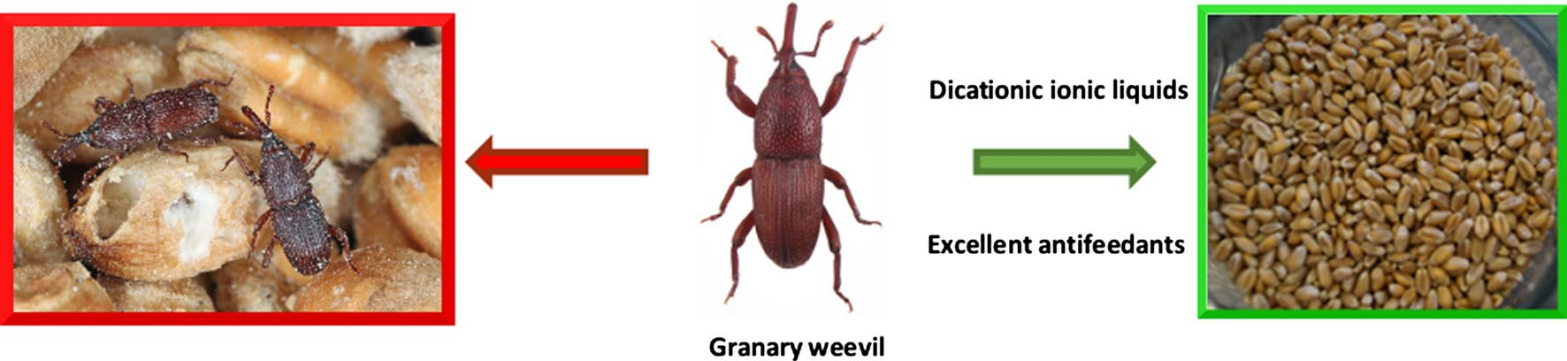

**Electronic supplementary material:**

The online version of this article (10.1007/s11696-018-0495-6) contains supplementary material, which is available to authorized users.

## Introduction

The use of synthetic insecticides for stored-product protection from pests has led to problems such as disturbances of the environment, insect resistance to pesticides and lethal effects on non-target organisms (Geng et al. 2011). It is necessary to develop safe and cheap methods for fighting harmful insects and searching for new active compounds, especially of a natural origin. Substances that deter or inhibit insect feeding are called food deterrents or antifeedants (Isman 2002). Due to their specific properties, insect antifeedants are environmentally friendly crop protection agents. Antifeedants are allomone substances that mainly inhibit feeding on stored products, limit the potential of pest development and do not kill them directly (Lozowicka and Kaczyński 2013). A key characteristic of these compounds is selective action. Antifeedants exhibit activity against parasites and do not cause any negative effects for useful insects such as pest predators or pollinators (Pavela 2010). In addition, the use of deterrents may limit the application of toxic insecticides and reduces the risk of contamination of food with dangerous compounds.

Dicationic ionic liquids (DILs) are a group of compounds consisting only of ions, they have a melting point of less than 100 °C. DILs are composed of two cations linked with a rigid or flexible linker and two anions. In the case of dicationic ionic liquids, the modification potential is much greater than in the case of monocationic ionic liquids. It is not only possible to select cations and anions, but also the type and length of the spacer (Claros et al., 2010, 2012). Additionally, DILs may contain appropriate functional groups, such as thiol, ether, hydroxyl and amino groups in the structure of cations (Lohar et al. 2016).

Currently, beside DILs, containing two imidazolium groups as cations, an examination of compounds with phosphonium (Yonekura and Grinstaff, 2014), cholinium (Silva et al. 2014), piperidinium (Haddad et al. 2014), pyridinium (Mahrova et al. 2015), cations as well as asymmetric DILs consisting of two different cations was also performed (Wang et al. 2015).

In contrast to monocationic ionic liquids with a single cation, DILs are characterized by higher density, viscosity and glass transition temperature. They also exhibit very high thermal stability. (Shirota et al. 2011; Claros et al. 2010). Low volatility and excellent thermal properties allow for their use in processes carried out in high temperatures. (Fan et al. 2013).

DILs can be applied as acidic (Azizi and Shirdel 2016) or basic catalysts (Fan et al. 2013), anti-corrosive additives and lubricants (Gindri et al. 2016a, b), antimicrobial agents (Cancemi et al. 2017) and coatings or antioxidants (Czerniak and Walkiewicz 2017).

The use of natural food deterrents would be the best solution due to ecological reasons. However, the cost of obtaining them is high and uneconomical. This cost limits the commercial application of natural compounds such as azadirachtin. Currently, research for new compounds with similar or better efficacy is being conducted.

Ionic liquids are used as cheap and safe feeding deterrents. These are mainly compounds containing ammonium cations such as didecyldimethylammonium. The presence of long alkyl chains determines the high activity of ionic liquids in relation to different types of beetles and pest larvae (Pernak et al. 2013).

The most favorable direction for the development of the third generation of ionic liquids, which are compounds exhibiting biological activity, applies to the synthesis of salts from natural and low-toxicity materials. Antifeedants in the form of ionic liquids may contain natural anions, e.g., lactate (Cybulski et al. 2008), theophyllinate (Markiewicz et al. 2014), fatty acids (Pernak et al. 2015) or abietate (Klejdysz et al. 2016). In addition, compounds based on artificial sweeteners also show good deterrent activity (Pernak et al. 2012).

The aim of this study was the synthesis of a new compound with antifeedant activity and containing gemini surfactant as a cation. As per the literature data, compounds having one long alkyl chain exhibited better biological properties than compounds with a short alkyl chain (Pernak et al. 2013). Compounds with a low toxicity (artificial sweeteners and natural acids) were used as a counterion. This combination was created to obtain a salt with the desired physicochemical properties and an increased biological activity. The reason for this research was the need to find new, cheaper and more active antifeedants.

## Experimental

### Materials

*N,N*-Dimethyldecylamine (90%, Sigma-Aldrich), 1,4-dibromobutene (99%, Sigma-Aldrich), 1,6-dibromohexane (96%, Sigma-Aldrich), 1,8-dibromooctane (98%, Sigma-Aldrich), 1,10-dibromodecane (97%, Sigma-Aldrich), 1,12-dibromododecane (98%, Sigma-Aldrich), acesulfame K (≥ 99%, Sigma-Aldrich), l-lactic acid (85%, Sigma-Aldrich), l-pyroglutamic acid (≥ 99%, Sigma-Aldrich), saccharin sodium hydrate (99%, Alfa Aesar), potassium hydroxide (85%, POCH), methanol, dimethyl sulfoxide, acetonitrile, acetone, 2-propanol, ethyl acetate, chloroform, toluene and hexane were purchased from Sigma-Aldrich and used without further purification. Water was deionized by demineralizer HLP Smart 1000 (Hydrolab).

### Synthesis

Alkane-1,X-bis(decyldimethylammonium) dibromides were obtained by quaternization reaction between appropriate dibromo-alkane (0.1 mol) and decyldimethylamine (0.2 mol). The reactions were conducted in acetonitrile at 60 °C for 24 h. Next, the solvents were removed by vacuum evaporator and the product of reaction was mixed with ethyl acetate. After adding the solvent, dibromide bis(ammonium) precipitated as white solid and was isolated by filtration. The end product was dried under a reduced pressure at 70 °C for 24 h.

The next step was the synthesis of dicationic ionic liquids. The first method was based on the bromide anion exchange reaction (metathesis reaction) in methanol according to the method described earlier (Aher and Bhagat 2016). The reaction was carried out between bis(ammonium) dibromide (0.01 mol) and two molar equivalents of potassium salts of appropriate acids (l-lactic acid, l-pyroglutamic acid) or commercially available saccharin sodium and acesulfame potassium salts. The precipitated inorganic salt was removed from the mixture by filtration and the solvent was evaporated under a reduced pressure. To remove all by-product, bis(ammonium) salts were dissolved in acetone. The white solid bromide sodium or potassium precipitated and was removed by vacuum filtration. Finally, the product was dried under a reduced pressure at 55 °C for 24 h.

The second method was based on acid–base neutralization reaction according to the method described previously (Niemczak et al. 2015). Quaternary bis(ammonium) dihydroxide was obtained by the anion exchange reaction of dibromide bis(ammonium) with potassium hydroxide in methanol at room temperature. After neutralization of the obtained quaternary bis(ammonium) dihydroxide (0.01 mol) with appropriate acids (0.02 mol), solvent was removed by vacuum evaporator and the product was dissolved in acetone to precipitate other pollutants. The solid was recovered by filtration and the solvent in the mixture was evaporated by vacuum evaporator. The last step was drying under a reduced pressure at 55 °C for 24 h.

### Characterization methods

The melting point was examined on METTLER TOLEDO MP 90 melting point system. The solubility had been studied accordance to Vogel’s method. The solubility had been determined in ten selected solvents, such as water, methanol, DMSO, acetonitrile, acetone, 2-isopropanol, ethyl acetate, chloroform, toluene and hexane.

Refraction index was measured for obtained salts which were liquid at room temperature. This test was made on Abbe Rudolph Research Analytical J357 Automatic Refractometer and it was set in a range from 20 to 80 °C.

Viscosity has been examined by rotational viscometer Rheotec RC30-CPS. The measurement consisted of examining the change in viscosity with temperature (20–80 °C).

Density was determined using an automatic density meter DDM2911 with a mechanical oscillator method. The density of the samples (approx. 2.0 cm^3^) was measured with respect to temperature-controlled conditions with Peltier, from 20 to 80 °C. The apparatus used was calibrated using deionized water as the reference substance.

The solubility of the prepared salts was determined according to Vogel’s Textbook of Practical Organic Chemistry (A. I. Vogel and B. S. Furniss, Vogel’s Textbook of Practical Organic Chemistry, Longman, 4th edn, 1984). Different solvents were selected for the solubility test, such as water, methanol, DMSO, acetonitrile, acetone, ethyl acetate, chloroform, toluene, and hexane. The solubility test was developed to classify the ionic liquid into one of three groups for each solvent. The first group included ILs, which dissolved (0.1 g of IL) in 1 ml of solvent; these compounds were described as “high solubility”. The term “moderate solubility” refers to 0.1 g compounds that have dissolved in 2 or 3 ml of solvent. The last group included 0.1 g ILs that did not dissolve in 3 ml and identified the compounds as “low solubility”. Tests were conducted at 25 °C under ambient pressure.

^1^H and ^13^C NMR spectroscopic analyses were carried out on a Varian Mercury 300 spectrometer operating at 300 and 75 MHz. Elemental analyses were performed at the Adam Mickiewicz University, Poznan (Poland). The internal standard was used in the analysis of tetramethylsilane and the solvent deuterated chloroform (CDCl_3_). CHN elemental analyses were performed at A. Mickiewicz University, Poznan (Poland).

Thermal gravimetric analysis (TGA) was performed using a Mettler Toledo Star^e^ TGA/DSC1 unit (Leicester, UK) under nitrogen. Samples (2–10 mg) were placed in aluminum pans and heated from 30 to 450 °C at a heating rate of 10 °C min^−1^.

Thermal transition temperature was determined by differential scanning calorimetry (DSC), with a Mettler Toledo Stare DSC1 (Leicester, UK) unit under nitrogen. Samples (5–15 mg) were placed in aluminum pans and heated from 25 to 100 °C at a heating rate of 10 °C min^−1^ and cooled with an intracooler at a cooling rate of 10 °C min^−1^ to -100 °C and then heated again to 100 °C.

### Methods of the deterrent activity experiments

Tests on feeding activity of ionic liquids were done on three species of most important pests of storage grain: granary weevil (*Sitophilus granarius* (Linnaeus, 1758)), confused flour beetle (*Tribolium confusum* Jacquelin du Val, 1868) and khapra beetle (*Trogoderma granarium* Everts, 1898). The above-mentioned insects were reared in the laboratory in an incubator at 26 (±) 10 °C and 60 (± 5) % relative humidity on uncrushed wheat grain (granary weevil) and shredded products from wheat grain: flour, bran and others (confused flour beetle and khapra beetle).

The experiment was conducted under identical conditions in which insects were reared (temperature and relative humidity). The choice and no-choice tests were done. The wafer discs were done from wheat flour. Discs were 1 cm diameter, 1 mm thick and its average weight was about 15 mg. Prepared wafers were saturated by dipping in either methanol only (control) or 1% solution of compounds in methanol and then left to air dry for 30 min. After this, wafers were weighed and offered to insects on Petri dish:

- two discs previously only dipped in a solvent (control for experiment in the variant no-choice test);
- two discs, one previously dipped in a solvent and second in 1% solution of tested compound (choice test);
- two discs previously dipped in a 1% solution of tested compound (no-choice test).

Both variants of experience and control were repeated five times. Experiments were performed on three species of insect: 3 adults of *S. granarius*, 20 adults and 10 larvae of *T. confusum* and 10 larvae of *T. granarium*. The number of insects used to experiment depended on the intensity of their food consumption. Adults used for experiments were unsexed, 7–10 days old, and the larvae were 5–30 days old. Insects were left in Petri dishes for 5 days, after this time, wafers were reweighed. Weight loss of the wafers was the basis to calculate three deterrency coefficients: *R* relative, *A* absolute and *T* total:

1 $$R = \frac{(C - E)}{(C + E)} \times 100\, \left( {\text{choice test}} \right)$$

2 $$A = \frac{{({\text{CC}} - {\text{EE}})}}{{({\text{CC}} + {\text{EE}})}} \times 100\, \left( {{\text{no}} - {\text{choice test}}} \right)$$

*C*, CC is the amount of food from the control discs consumed.

*E*, EE is the amount of food treated with tested compound consumed.

Total coefficient of deterrence (*T*) was the sum of relative and absolute coefficient.

The total coefficient of deterrence, which ranged from – 200 to 200, served as the index activity. The compounds with *T* values ranging from 151 to 200, are very good deterrents, those with coefficients values 101–150 are good deterrents and for the medium active *T* ranged from 51 to 100. Compounds with *T* values lower than 50 are weak deterrents. The coefficients of deterrence of group of compounds were analyzed by means of one-way ANOVA followed by the post hoc Tukey test with homogenous subset. Calculations were performed using Statistica 6.0.

## Results and discussion

**Scheme 1:**
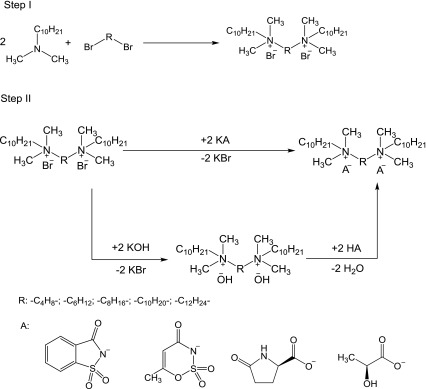
Synthesis of dicationic ionic liquids

In our work, five new bis(ammonium) dibromides were synthesized (Scheme 1, Step I) with a yield of about 90%. The purity of compounds was measured and confirmed by NMR. The melting points of salts are presented in Table 1.

**Table 1:** Bis(ammonium) dibromide salts

Salt	R	Yield (%)	Melting point (°C)
**1**	–C_4_H_8_–	95	218–221
**2**	–_6_H_12_–	92	235–236
**3**	–C_8_H_16_–	93	174–178
**4**	–C_10_H_20_–	90	124–127
**5**	–C_12_H_24_–	91	103–105

The next step was the synthesis of a compound with deterrent activity. This compound was obtained in an exchange reaction (Scheme 1, Step II). As a result of this reaction, 20 new salts were created (Table 2).

**Table 2:** Dicationic ionic liquids

Salt	Anion	Yield (%)	State at 25 (°C)	Melting point (°C)
**1a**	Saccharinate	89	Grease	–
**2a**		70	Solid	116.2–166.8
**3a**		96	Solid	75.2–75.6
**4a**		92	Solid	106.6–106.3
**5a**		91	Solid	121.1–121.9
**1b**	Acesulfamate	81	Grease	–
**2b**		83	Solid	102.6–102.8
**3b**		85	Solid	141.0–141.4
**4b**		89	Grease	–
**5b**		82	Grease	–
**1c**	Lactate	90	Liquid	–
**2c**		86	Grease	–
**3c**		99	Grease	–
**4c**		90	Liquid	–
**5c**		90	Liquid	–
**1d**	Pyroglutamate	75	Grease	–
**2d**		70	Grease	–
**3d**		90	Grease	–
**4d**		95	Grease	–
**5d**		99	Grease	–

The synthesis of ILs was conducted with two methods with a yield between 70 and 99%. All salts with cation hexamethylene-1,6-bis(dimethyldecylammonium) (**2a**, **2b**, **2c**, **2d**) had the lowest efficiency. Three salts were liquid at room temperature and eleven were greasy with high viscosity and density. Fifteen of the obtained salts can be included as ionic liquids.

**Fig. 1:**
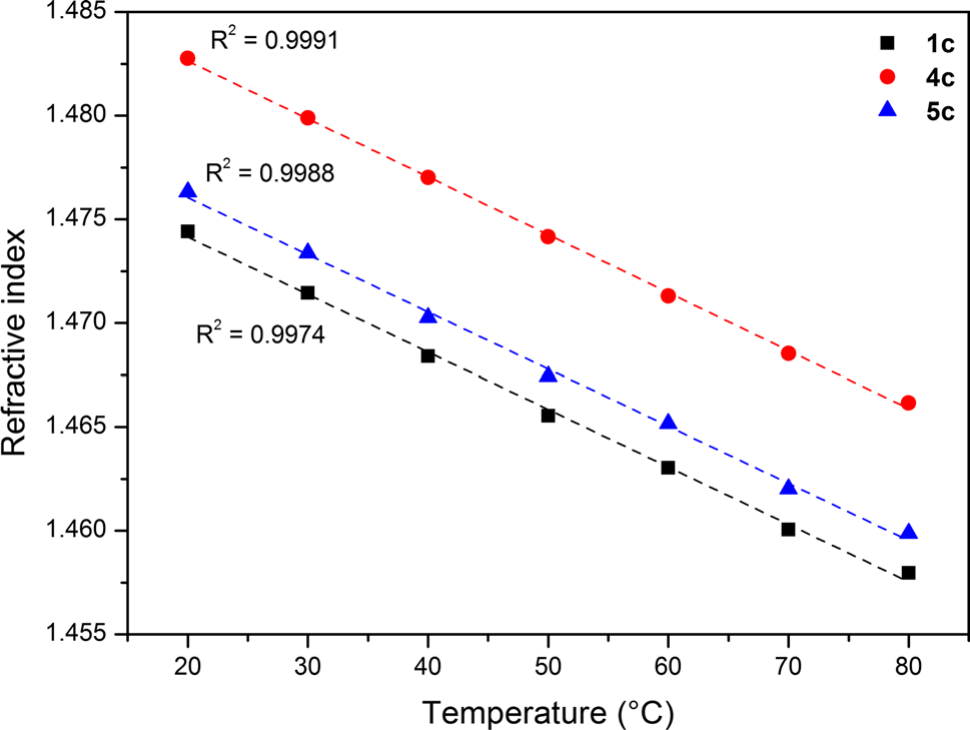
Refractive index of obtained RTILs (**1c**, **4c**, **5c**)

The impact of the anion on the chemical shifts of the alkyl chain was not significant in the analysis of the proton nuclear magnetic resonance. The shifts for hydrogen atoms at the last and penultimate carbon atom in the alkyl chain (–C_10_H_21_) are, respectively, 0.88 and 1.26 ppm, The CH_2_ groups had the lowest chemical shift for saccharinate anion, but the highest one for pyroglutamate anion. Chemical shifts of the atoms next to the quaternary nitrogen atom strongly depend on the anions. Compounds with lactate and pyroglutamate anions had the lowest values of chemical shifts, and the saccharinate anions and acesulfamate anions caused a shift towards a higher value. The viscosity, density and refractive index were examined for DILs which were liquid at 20 °C (RTILs). Figure 1 shows refractive index of obtained RTILs.

**Fig. 2:**
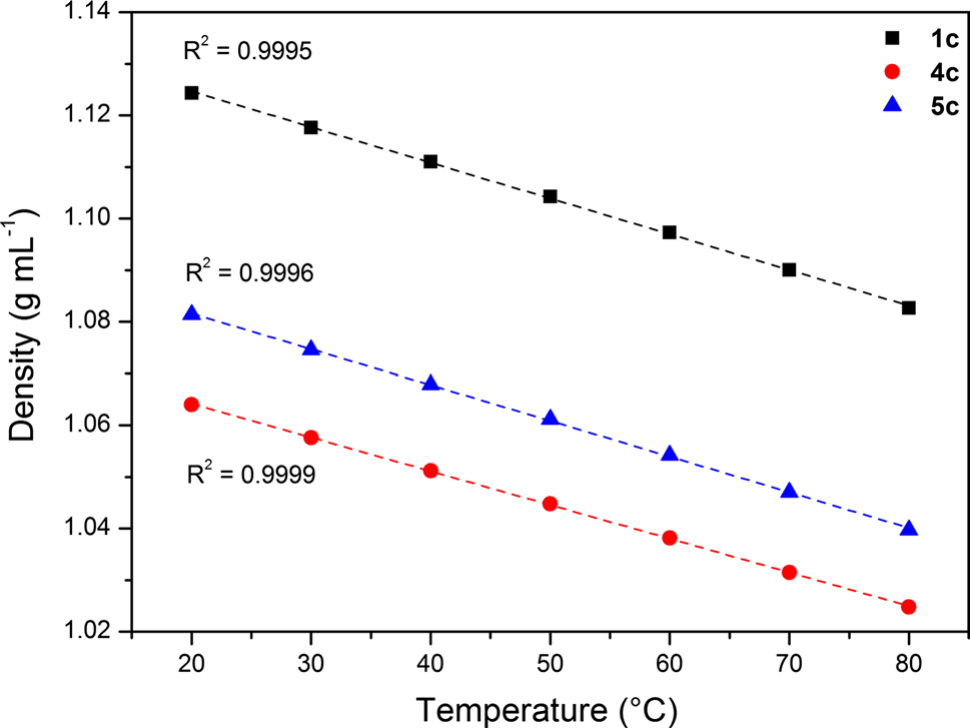
Density of synthesized salts **1c**, **4c** and **5c**

When analyzing the impact of temperature on the refraction index for three ionic liquids with a lactate anion (**1c**, **4c** and **5c**), a linear relationship was observed. The highest refraction index was observed for **4c** in the entire temperature range. In addition, a linear relationship between density and temperature was also noted (Fig. 2).

Compound **1c** had the highest density. The density of liquids **4c** and **5c** was almost the same as water. In the literature, data for single ionic liquids, the correlation between refractive index and the density were described. From these data, it follows that ionic liquids with the highest refractive index also had the highest values of density. In the case of the examined ionic liquids, an inverse relationship was observed. The lowest measured density values were recorded for the highest refractive index values.

**Fig. 3:**
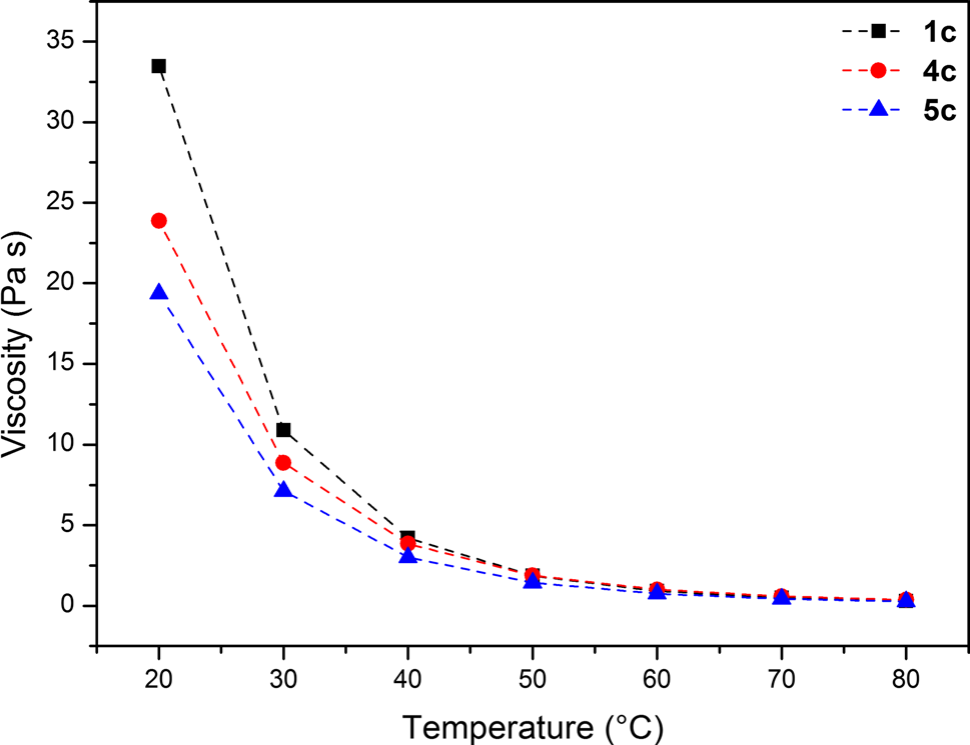
Viscosity of synthesized ionic liquids **1c**, **4c** and **5c**

Figure 3 shows the temperature dependence of the viscosity for dilactate alkyl-1,X-bis(decyldimethylammonium). Viscosity was measured for compounds **1c**, **4c** and **5c**. ILs **1c** had the highest viscosity; on the other hand, liquid **5c** had the lowest viscosity. Two intervals were observed. In the first interval, for a temperature of 20–40 °C, there is a sharp decrease in viscosity with a rise of temperature. In contrast, in the second interval (40-80 °C), a decrease in the measured viscosity value is much lower with an increase in temperature. Such a dependence is also observed for single ionic liquids with long alkyl chains.

Table 3 presents solubility of synthesized salts. All of them were insoluble in nonpolar solvents such as ethyl acetate, toluene and hexane, but soluble in chloroform.

**Table 3:** Solubility of prepared salts at 25 °C

Salt	Anion	Solvents									
		A	B	C	D	E	F	G	H	I	J
**1a**	Saccharinate	±^b^	+^a^	±	+	+	+	−^c^	+	−	−
**2a**		−	+	±	+	+	+	−	+	−	−
**3a**		−	+	±	+	+	+	−	+	−	−
**4a**		−	+	±	+	+	+	−	+	−	−
**5a**		−	+	±	±	+	±	−	+	−	−
**1b**	Acesulfamate	±	+	−	+	+	+	−	+	−	−
**2b**		−	+	−	+	+	±	−	+	−	−
**3b**		−	+	−	+	+	±	−	+	−	−
**4b**		−	+	−	+	+	±	−	+	−	−
**5b**		−	+	−	+	+	±	−	+	−	−
**1c**	Lactate	±	+	±	−	+	−	−	+	−	−
**2c**		±	+	±	−	±	−	−	+	−	−
**3c**		±	+	±	−	±	−	−	+	−	−
**4c**		±	+	±	−	±	−	−	+	−	−
**5c**		±	+	±	−	±	−	−	+	−	−
**1d**	Pyroglutamate	±	+	±	−	±	−	−	+	−	−
**2d**		+	+	±	−	±	−	−	+	−	−
**3d**		+	+	+	−	±	−	−	+	−	−
**4d**		+	+	+	−	±	−	−	+	−	−
**5d**		+	+	+	−	±	−	−	+	−	−

*A* water, *B* methanol, *C* DMSO, *D* acetonitrile, *E* acetone, *F* isopropanol, *G* ethyl acetate, *H* chloroform, *I* toluene, *J* hexane

^a^High solubility, ^b^moderate solubility, ^c^low solubility

Salts with saccharin anions (**1a**–**5a**) were soluble in polar solvents, except for water. Synthesized salts with acesulfame anions (**1b**–**5b**) were characterized by weaker solubility in DMSO and isopropanol in comparison to **1a–5a** salts. Compounds **1b**–**5b** were insoluble in DMSO and dissolved poorly in isopropanol.

The salts with lactate and pyroglutamate anions were insoluble in solvents such as isopropanol and acetonitrile, but exhibited solubility in methanol and water. However, compounds **2d**–**5d** dissolve better than salts **1c**–**5c** and **1d**. DILs **1c**–**5c** and **1d**–**5d** dissolved poorly in acetone and acetonitrile, except for **3d**–**5d**.

Thermal stability and differential scanning calorimetry tests were conducted for all synthesized salts. The results of the studies are presented in Table 4.

**Table 4:** Thermal analysis (DCS and TG) of synthesis salts

Salt	T_g_ (°C)	T_c_ (°C)	T_m_ (°C)	T_onset5%_ (°C)	T_onset50%_ (°C)
**1a**	5.6	–	–	215	251
**2a**	2.7	–	–	222	256
**3a**	− 4.5	54.7	83.4	224	265
**4a**	4.8	–	–	222	268
**5a**	− 5.5	–	–	236	320
**1b**	6.0	–	–	222	273
**2b**	− 5.0	62.7	83.1	230	284
**3b**	14.5	–	–	226	270
**4b**	− 21.3	–	–	230	288
**5b**	–	–	–	225	271
**1c**	− 26.7	–	–	200	237
**2c**	− 29.2	–	–	207	249
**3c**	− 34.9	–	–	210	243
**4c**	− 30.1	–	–	210	257
**5c**	− 32.9	–	–	211	262
**1d**	− 25.1	–	–	198	253
**2d**	− 39.0	–	–	214	257
**3d**	− 15.3	–	–	193	265
**4d**	− 12.5	–	–	213	272
**5d**	− 11.1	–	–	214	284

*T*_*g*_ glass transition temperature, *T*_*c*_ temperature of crystallization, *T*_*m*_ melting point, *T*_*onset5%*_ decomposition temperature of 5% sample, *T*_*onset50%*_ decomposition temperature of 50% sample

It was observed that salts with acesulfamate and saccharinate anions have lower values of glass transition temperature (*T*_g_) than pyroglutamate or lactate anions. The lowest glass transition temperature for **1c**–**5c** was recorded for an eight-carbon alkyl linker and the highest for ILs with four carbon atoms in the linker. In the case of the series with a pyroglutamate anion, compounds with the shortest linker had a very low *T*_g_, while the **3d–5d** DILs had a similar *T*_g_ and ranged from − 11.1 °C (**5d**) to − 15.3 °C (**3d**). Salts with saccharin anions had the highest glass transition temperature in the range from − 5.5 °C (**5a**) to 5.6 °C (**1a**). Only two compounds (**3a** and **2b**) exhibited a temperature of crystallization (*T*_c_) and a melting point (*T*_m_) in the tested temperature range.

The thermal stability of the tested salts was also determined. It can be seen that compounds with saccharinate anions (**1a–5a**) and acesulfamate (**1b–5b**) have a slightly higher stability than the other synthesized salts. DILs **1d–5d** were characterized by a decrease in the *T*_onset5%_ value for ILs with eight carbon atoms in the alkyl linker (**3d**). *T*_onset_ values ranged from 237 to 320 °C. The lowest values were recorded for the **1c–5c** series, where the minimum value was observed in the case of **3c** DIL. On the other hand, the highest values were observed for compounds with the acesulfamate anion, except for **5b**, where a decrease in the *T*_onset50%_ parameter was noted. The other two series (**1a–5a** and **1d–5d**) were characterized by an almost linear increase in stability with growth in the alkyl linker, except for DIL **5a**.

Compounds with a lactate anion and the didecyldimethylammonium (DDA) cation described in the literature showed higher thermal stability, but the ones with benzyldimethylalkyl ammonium (BA) cation exhibited a lower value than in the case of all the obtained salts. In addition, compounds with DDA and BA cations had a glass transition temperature, which was not observed in bis(ammonium) compounds.

**Fig. 4:**
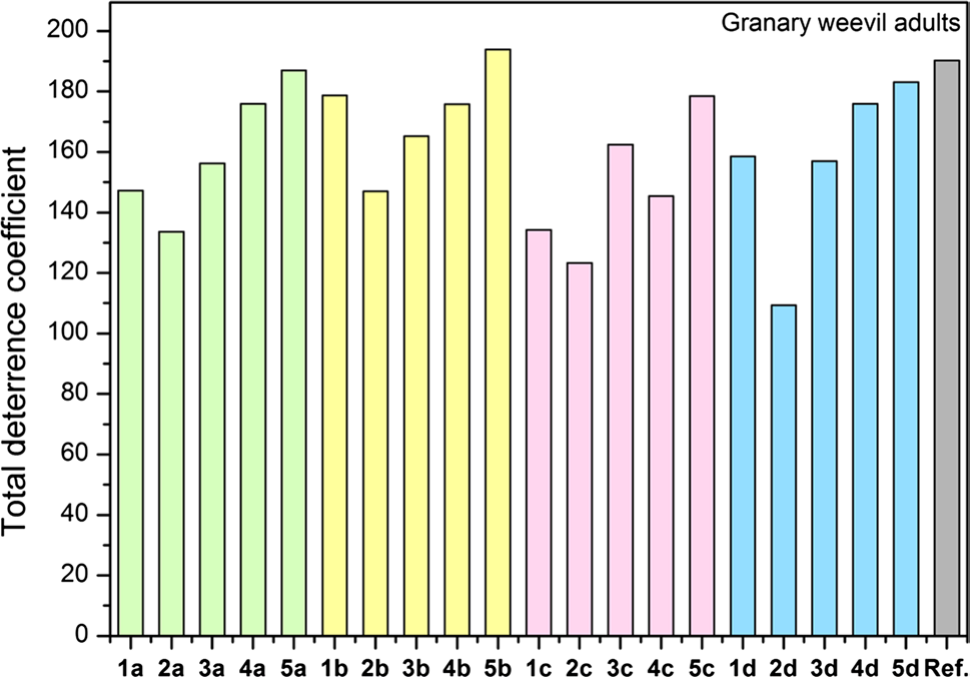
Deterrent activity towards granary weevil (*Sitophilus granaries*)

ILs with cationic species such as chlormequat (CC), diallyldimethylammonium (DADMA), didecyldimethylammonium (DDA), hexadecylpyridinium (HEXA) or benzylalkyldimethylammonium (BA), acesulfame and saccharinate anions have already been reported. Compounds with CC and DADMA cations exhibited higher thermal stability than the synthesized compounds, but in the case of DDA, HEXA and BA, the stability was lower or comparable with the tested substances. Antifeedants containing anion deriving from natural acids have also been reported in the literature. These compounds exhibited thermal stability at a similar level as bis(ammonium) compounds or slightly higher (Pernak et al. 2013; Cybulski et al. 2008; Hough-Troutman et al. 2009). DILs with lactate, pyroglutamate, saccharinate and acesulfamate anions were stated by very good or good deterrent properties (Fig. 4).

In the case of granary weevil (*Sitophilus granaries*), the synthesized series of quaternary ammonium compounds with acesulfamate, saccharinate and pyroglutamate anions had minimum activity for a compound with six carbon atoms in the linker alkyl.

**Fig. 5:**
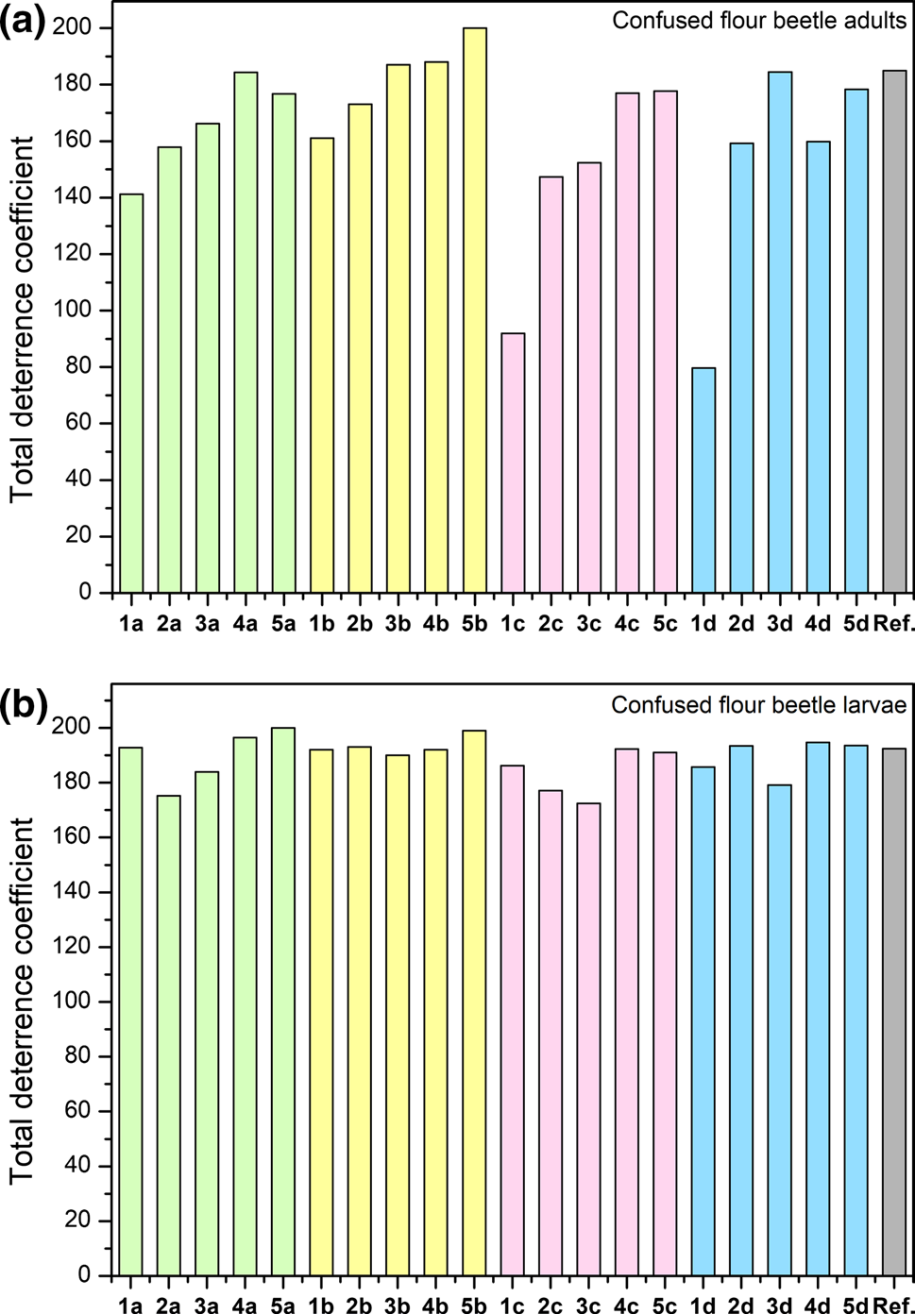
Deterrent activity towards adult and larvae confused flour beetle (*Tribolium confusum*)

Statistical analysis of the deterrent’s activity confirmed very good antifeedant properties for all synthesized compounds with eight, ten and twelve carbon atoms in the linker alkyl, beyond the series with lactate anions (**1c–5c**). The same activity was presented by salts with an acesulfamate and pyroglutamate anion and four carbon atoms in the linker (Fig. 5).

**Fig. 6:**
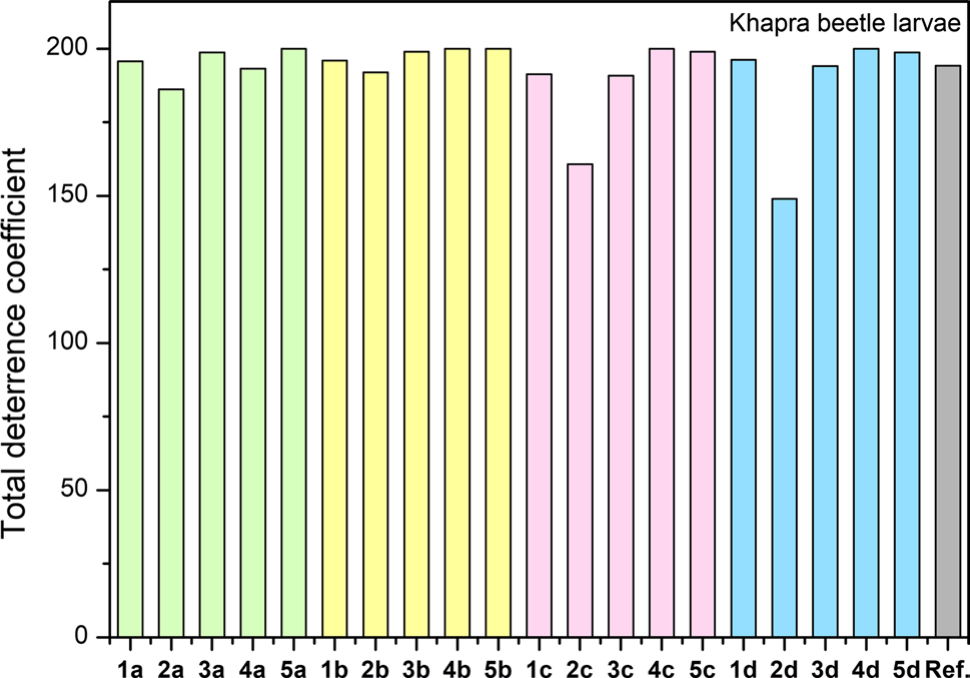
Biological activity of synthesized ionic liquids towards khapra beetle (*Trogoderma granarium*)

In the case of an adult confused flour beetle (*Tribolium confusum*), all salts with acesulfamate anions (**1b–5b**) showed activity at a very good level. This series noted a linear increase with an extension linker between quaternary nitrogen. A similar relationship was observed for salts with saccharinate anions (**1a–5a**). The rest of the compounds exhibited the lowest activity for the shortest linker. Synthesized ILs can be included in three groups with medium, good and very good activity. Activity towards an adult confused flour beetle increased with the number of carbon atoms with the exception of salts with a pyroglutamate anions (**1d–5d**). The graph of larvae confused flour beetle (*Tribolium confusum*) showed similar activity for all salts obtained, at a very good activity level (Fig. 6).

Obtained ILs showed very good activity towards larvae khapra beetle (*Trogoderma granarium*). From each series of compounds, the lowest activity was noted for the salts with a linker with six carbon atoms (**2c**, **3d**). Taking into account the data describing the biological activity for all the obtained compounds towards tested insects, the slight advantage of the salt with the cation long alkyl linkers (in particular for the linker with 12 carbon atoms) was noticeable, but there was no such dependence for all tested insects. ILs with 8, 10 or 12 carbon atoms in the bis(ammonium) cation were characterized by the highest activity than the reference compound (Azadirachtin) to insects such as the confused flour beetle or larvae of both tested species.

On the basis of previous data regarding ILs with acesulfamate, saccharinate and lactate anions, it can be concluded that synthesized DILs have high feeding deterrence activity.

Efficacy of salts with acesulfamate and saccharinate anion depends most likely on the amphiphilicity of the cation. Hence, acesulfamates and saccharinates comprising [2-(acryloyloxy)ethyl]trimethylammonium, [2-(methacryloyloxy)ethyl]trimethylammonium, 2-chloroethyltrimethylammonium, trimethylvinylammonium or (2-acetoxyethyl)alkoxymethyl-dimethylammonium cations were characterized by medium or weak biological activity (*T* coefficient did not exceed 114). However, antifeedant activity for ILs with more hydrophobic cations was significantly higher, and in several cases (i.e., alkoxymethyl(2-hydroxyethyl)dimethylammonium, didecyldimethylammonium salts) exhibited very good efficacy similar to the reference substance (azadirachtin—the compound of natural origin with the highest feeding deterrence activity). The feeding deterrence activity was determined also for precursor of ILs with didecyldimethylammonium cation—didecyldimethylammonium chloride. The obtained results allowed us to evaluate the impact of anion exchange on antifeedant activity—saccharinates or acesulfamates were characterized by increased biological activity in comparison to precursor with chloride anion. Therefore, we can conclude that used bis(ammonium) dibromides also have a deterrent activity. Furthermore, in terms of general efficacy against all of the tested insects, the obtained DILs exhibit the best efficiency (Czerniak et al. 2014; Pernak et al. 2013; Hough-Troutman et al. 2009; Pernak et al. 2007).

According to the previous papers, the highest deterrent activity (*T* parameter equal to 190) was reached for the IL with lactate anion. The obtained DILs with the same anion exhibited the highest possible efficacy according to the methodology (*T* equal to 200)—a noticeably better result in comparison to azadirachtin (Cybulski et al. 2008; Czerniak et al. 2014). So far, no studies on the feeding deterrence of the ILs with pyroglutamate anion have been reported.

## Conclusions

Two methods of synthesis of bis(ammonium) salt and ionic liquids with sweet anions (acesulfamate, saccharinate), as well as anions occurring naturally (lactate and pyroglutamate) and gemini surfactant cations were described. The selection of cation and anion allowed to design the desired properties of the obtained compounds.

The effect of the anion and length of the alkyl linker on solubility was determined. The compounds with sweet anions had medium or weak solubility in water, in comparison to compounds with natural anions. An inverse correlation in solubility for solvents such as acetonitrile and isopropanol was observed. In addition, the influence of an alkyl linker on physicochemical properties such as density, viscosity and refractive index was determined for bis(ammonium) lactates. An important advantage of the synthesized compounds was high thermal stability (values ranging from 237 to 320 °C), which also allowed them to be exposed to high-temperature conditions.

Furthermore, it was noted that most of the salts obtained had a comparable or better antifeedant activity than azadirachtin. The highest efficacy towards all examined storage insects was observed for compounds with twelve carbon atoms in the alkyl linker.

## Electronic supplementary material

Below is the link to the electronic supplementary material.

Supplementary material 1 (DOCX 2109 kb)
