# Supplementary material for: Dicationic ionic liquids as new feeding deterrents
Source: Chem Zvesti. 2018 May 16;72(10):2457–66. doi: 10.1007/s11696-018-0495-6 (PMC6096682; doi:10.1007/s11696-018-0495-6)
Supplement: Supplementary file 1 — Supplementary material 1 (DOCX 2109 kb) [file 11696_2018_495_MOESM1_ESM.docx]

Electronic Supporting Information (ESI)

Dicationic ionic liquids as new feeding deterrents

**damian k. kaczmarek^*1^, Kamil Czerniak^1^, Tomasz Klejdysz^2^**

^1^ Department of Chemical Technology, Poznan University of Technology, 60-965 Poznan, Poland

^2^ Institute of Plant Protection-National Research Institute, 60-318 Poznan, Poland,

**^*^**Corresponding author, e-mail: damian.rom.kaczmarek@doctorate.put.poznan.pl

tetramethylene-1,4-bis(decyldimethylammonium) disaccharinate (**1a**)

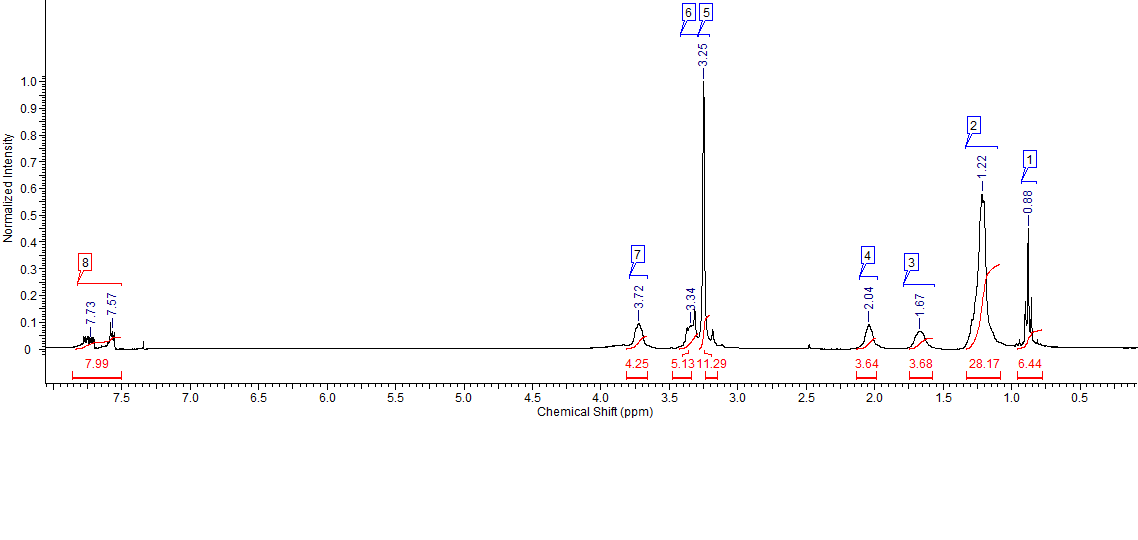


^1^H NMR (CDCl_3_) δ [ppm] = 0.88 (m. *J*=7.28 Hz. 6H): 1.22 (m. 28H): 1.67 (s. 4H): 2.04 (s. 4H): 3.25 (s. 12H): 3.34 (s. 4H): 3.72 (s. 4H): 7.57 (m. 4H): 7.73 (m. 4H):

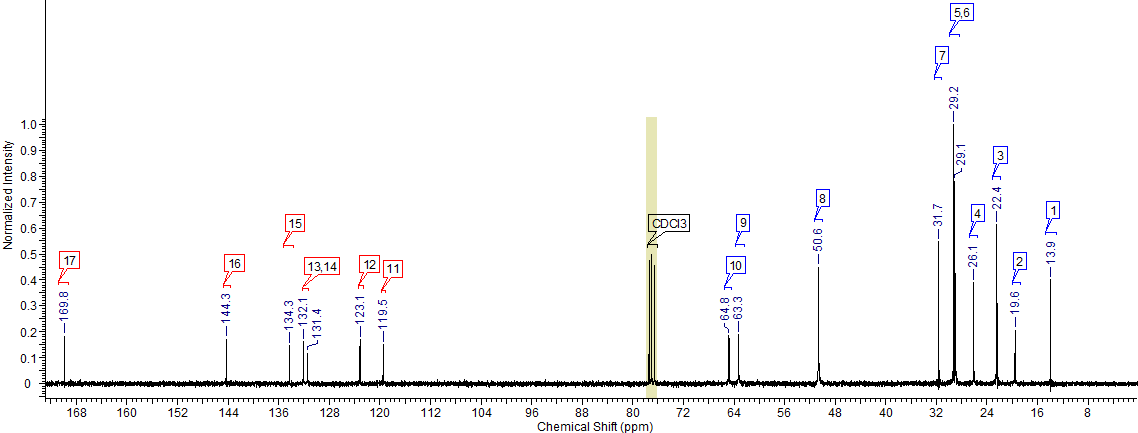


^13^C NMR (CDCl_3_) δ [ppm] = 13.9 [2C]: 19.6 [2C]: 22.4 [2C]: 26.1 [4C]: 29.1 [4C]: 29.2 [4C]: 31.7 [2C]: 50.6 [4C]: 63.3 [2C]: 64.8 [2C]: 119.5 [2C]: 123.1 [2C]: 131.4 [2C]: 132.1 [2C]: 134.3 [2C]: 144.3 [2C]: 169.8 [2C].

Elemental analysis CHN for C_42_H_70_N_4_O_6_S_2_ (Mmol = 791.16 g/mol): calculated values (%):
C = 63.76; H = 8.92; N = 7.08; measured values (%): C = 63.36; H = 8.61; N = 7.41.

hexamethylene-1,6-bis(decyldimethylammonium) disaccharinate (**2a**)


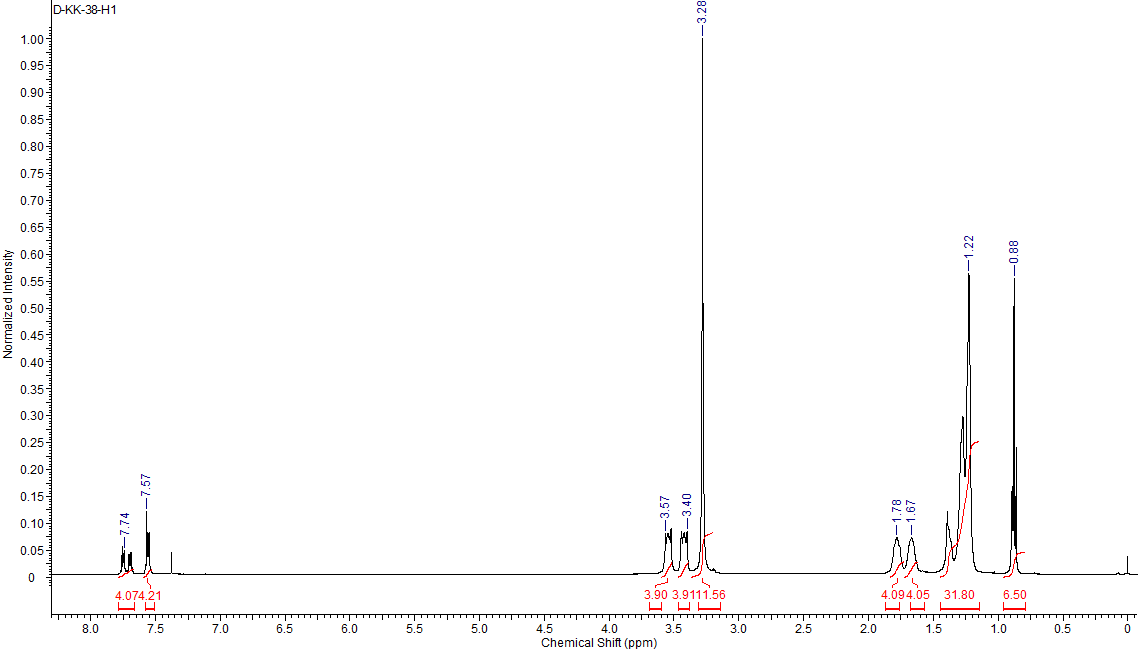


^1^H NMR (CDCl_3_) δ [ppm] = 0.88 (m. *J*=7.07 Hz. 6H): 1.22 (m. 32H): 1.67 (s. 4H): 1.78 (s. 4H): 3.28 (s. 12H): 3.40 (s. 4H): 3.57 (s. 4H): 7.57 (m. 4H): 7.74 (m. 4H):


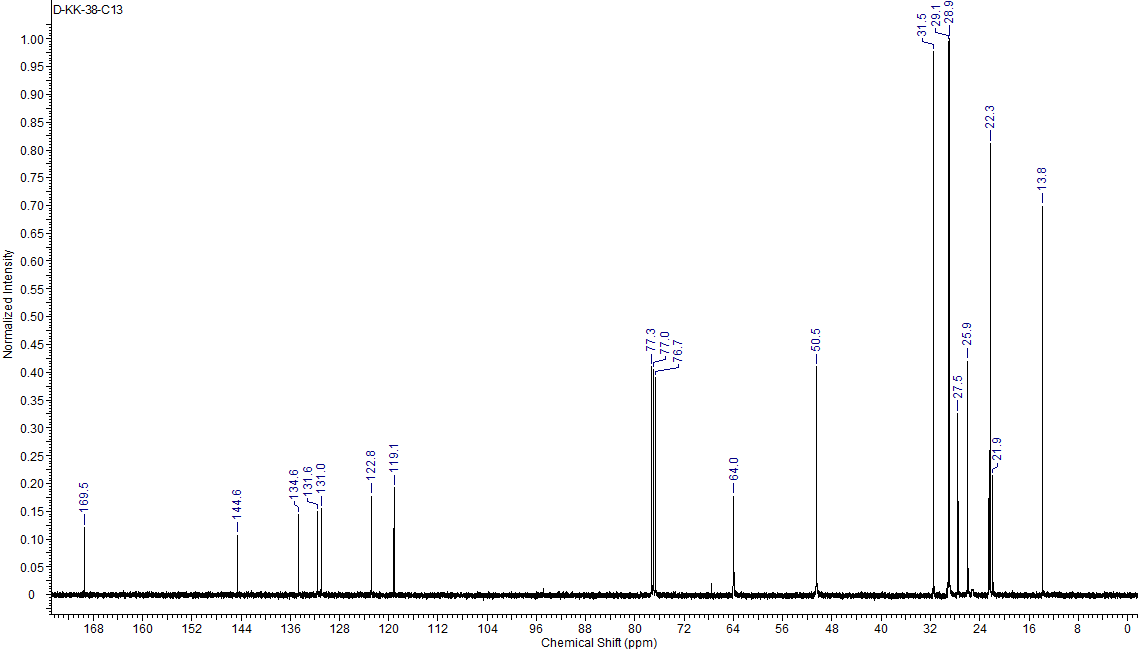


^13^C NMR (CDCl_3_) δ [ppm] = 13.8 [2C]: 21.9 [1C]: 22.3 [1C]: 25.9 [4C]: 27.5 [4C]: 28.9 [4C]: 29.1 [4C]: 31.5 [2C]: 50.5 [4C]: 64.0 [4C]: 119.1 [2C]: 122.8 [2C]: 131.0 [2C]: 131.6 [2C]: 134.6 [2C]: 144.6 [2C]: 169.5 [2C].

Elemental analysis calculated for C_44_H_74_N_4_O_6_S_2_ (Mmol = 819.22 g mol^-1^) (%): C = 64.51; H = 9.17; N = 6.84; found: C = 64.13; H = 9.55; N = 7.15.

octamethylene-1,8-bis(decyldimethylammonium) disaccharinate (**3a**)


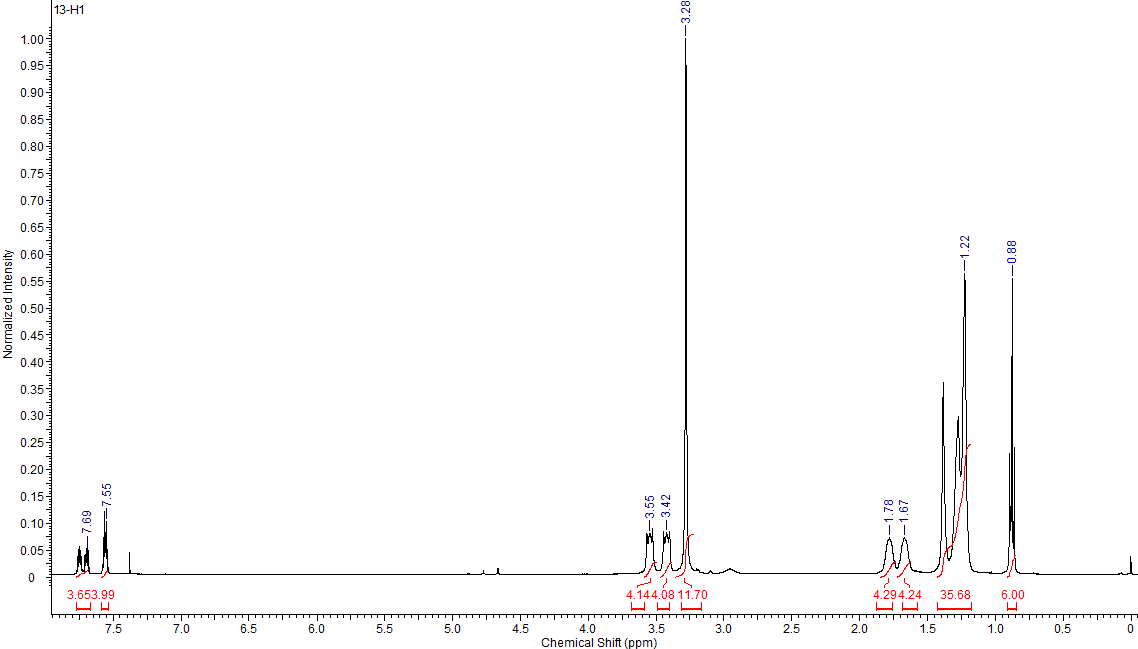


^1^H NMR (CDCl_3_) δ [ppm] = 0.88 (m. *J*=6.9 Hz. 6H): 1.22 (m. 36H): 1.67 (s. 4H): 1.78 (s. 4H): 3.28 (m. 12H): 3.42 (s. 4H): 3.55 (s. 4H): 7.55 (m. 4H): 7.69 (m. 4H):


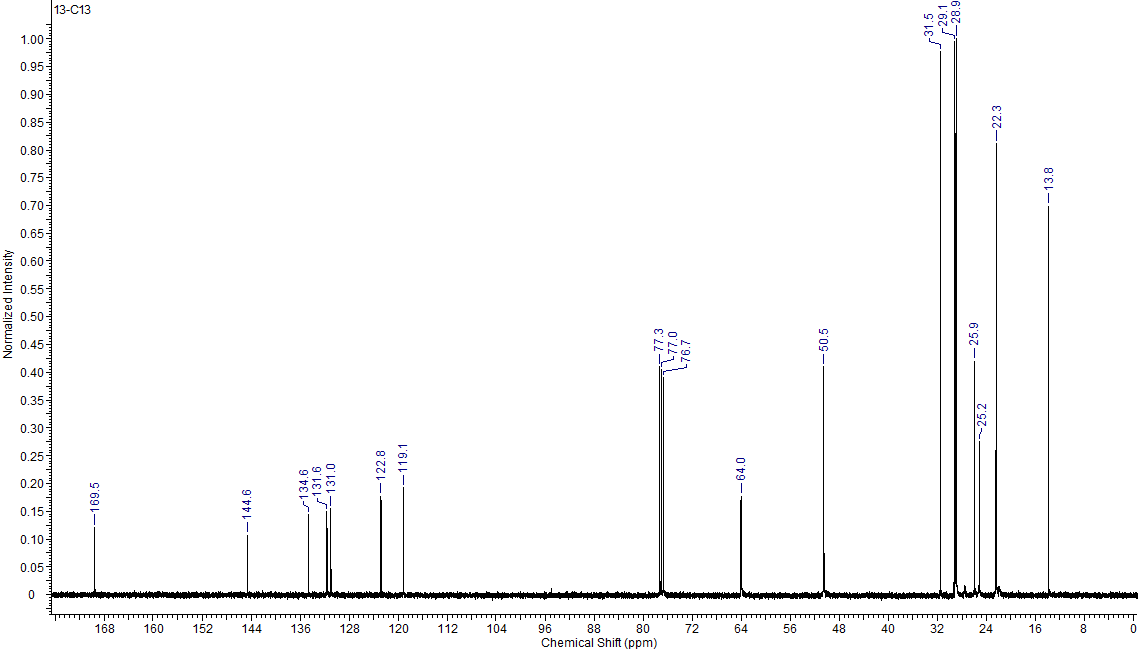


^13^C NMR (CDCl_3_) δ [ppm] = 13.8 [2C]: 22.3 [2C]: 25.2 [4C]: 25.9 [4C]: 28.9 [6C]: 29.1 [4C]: 31.5 [2C]: 50.5 [4C]: 64.0 [4C]: 119.1 [2C]: 122.8 [2C]: 131.0 [2C]: 131.6 [2C]: 134.6 [2C]: 144.6 [2C]: 169.5 [2C].

Elemental analysis calculated for C_46_H_78_N_4_O_6_S_2_ (Mmol = 847.27 g mol^-1^) (%): C = 65.21; H = 9.28; N = 6.61; found: C = 65.52; H = 9.60; N = 6.25.

decamethylene-1,10-bis(decyldimethylammonium) disaccharinate (**4a**)


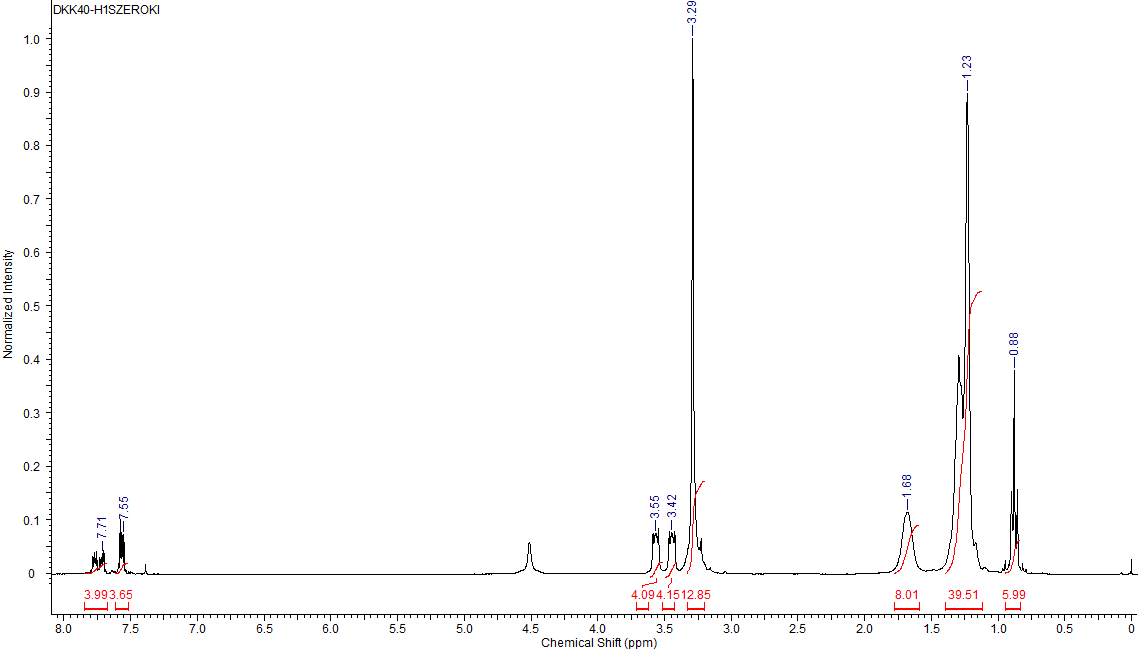


^1^H NMR (CDCl_3_) δ [ppm] = 0.88 (m. *J*=6.8 Hz. 6H): 1.23 (m. 40H): 1.67 (s. 8H): 3.30 (m. 12H): 3.42 (s. 4H): 3.55 (s. 4H): 7.57 (m. 4H): 7.73 (m. 4H):


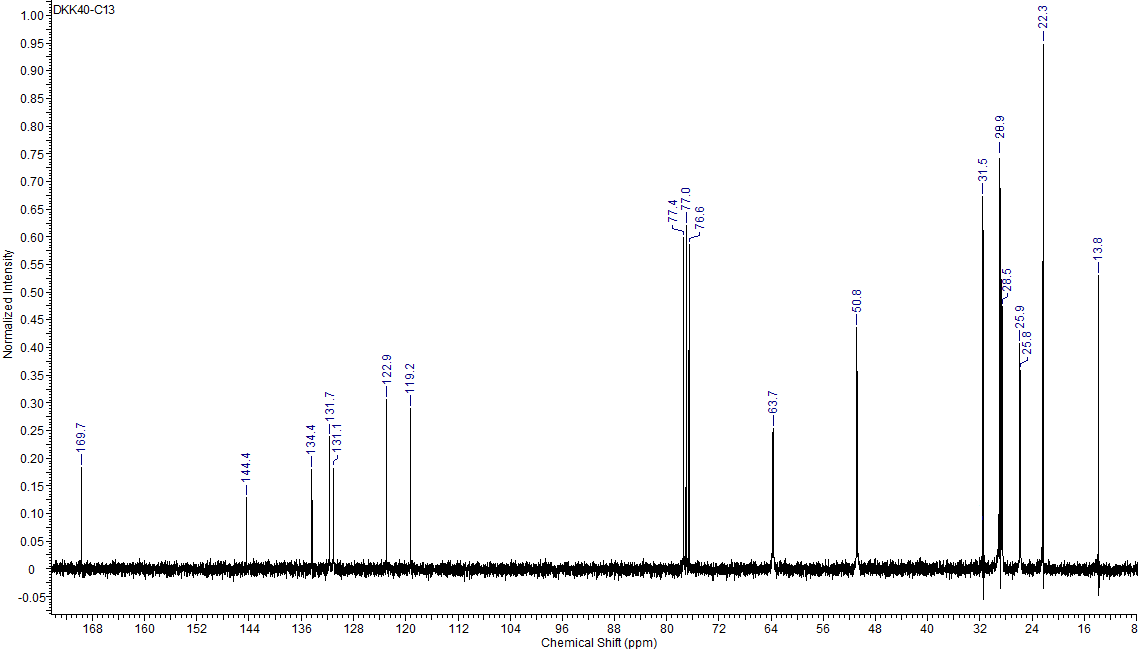


^13^C NMR (CDCl_3_) δ [ppm] = 13.8 [2C]: 22.3 [2C]: 25.8 [4C]: 25.9 [4C]: 28.5 [6C]: 28.9 [6C]: 31.5 [2C]: 50.8 [4C]: 63.7 [4C]: 119.2 [2C]: 122.9 [2C]: 131.1 [2C]: 131.7 [2C]: 134.4 [2C]: 144.4 [2C]: 169.7 [2C].

Elemental analysis calculated for C_48_H_82_N_4_O_6_S_2_ (Mmol = 875.33 g mol^-1^) (%): C = 65.86; H = 9.44; N = 6.40; found: C = 65.46; H = 9.10; N = 6.00.

dodecamethylene-1,12-bis(decyldimethylammonium) disaccharinate (**5a**)


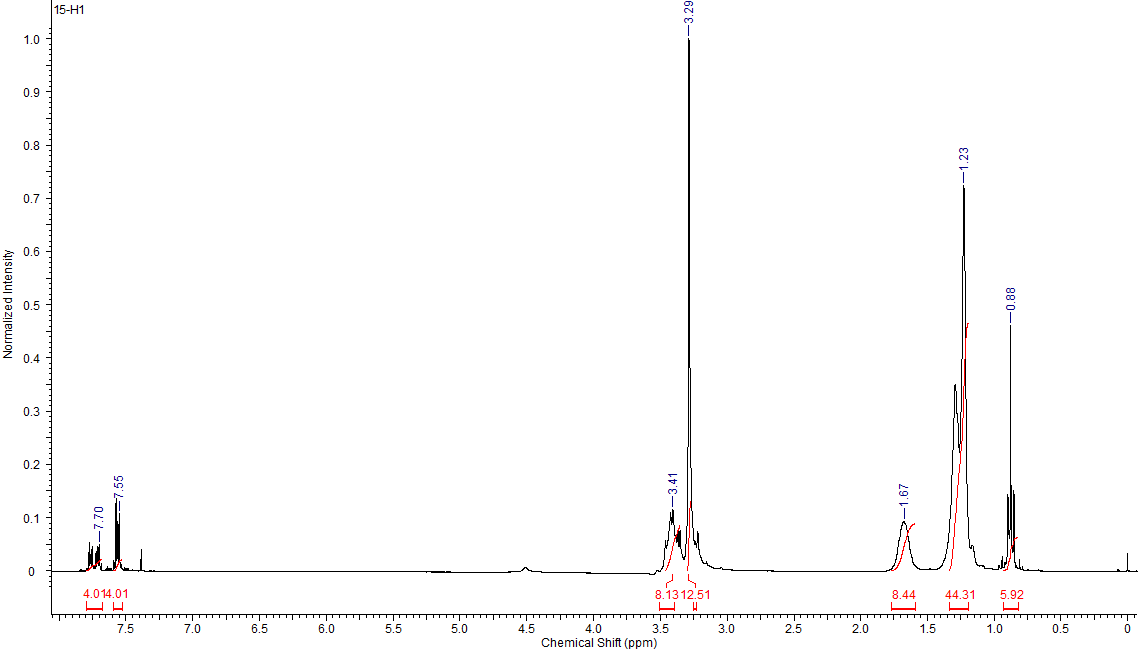


^1^H NMR (CDCl_3_) δ [ppm] = 0.88 (m. *J*=6.75 Hz. 6H): 1.23 (m. 44H): 1.67 (s. 8H): 3.29 (m. 12H): 3.41 (m. 8H): 7.55 (m. 4H): 7.70 (m. 4H):


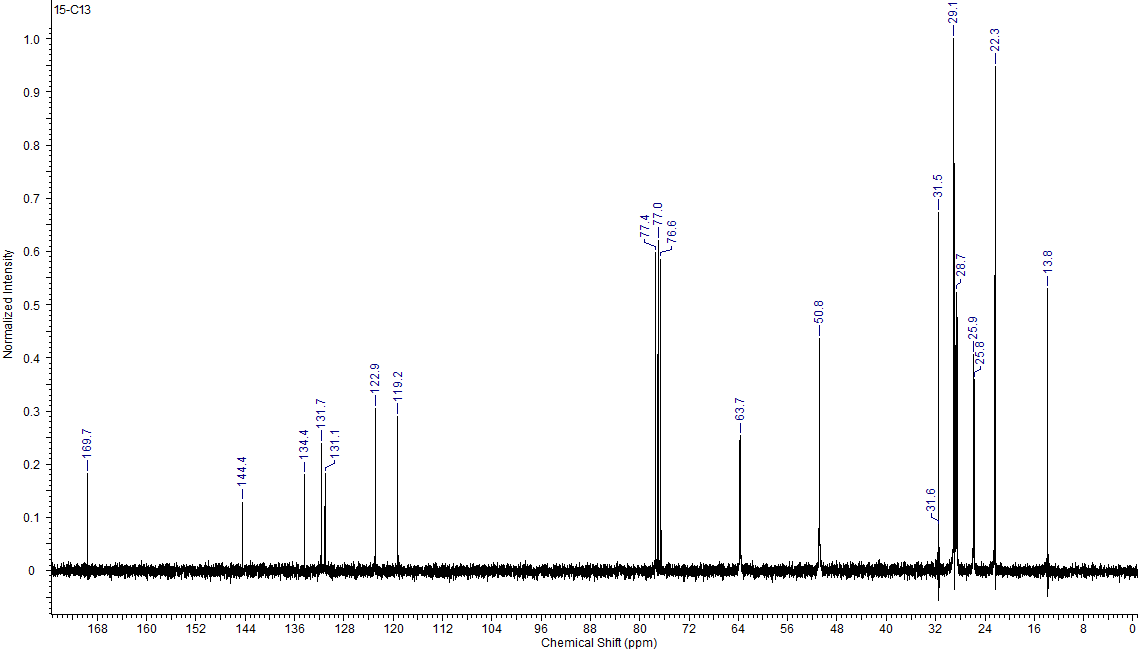


^13^C NMR (CDCl_3_) δ [ppm] = 13.8 [2C]: 22.3 [2C]: 25.7 [4C]: 25.9 [4C]: 28.7 [6C]: 29.1 [8C]: 31.5 [2C]: 50.8 [4C]: 63.7 [4C]: 119.2 [2C]: 122.9 [2C]: 131.1 [2C]: 131.7 [2C]: 134.4 [2C]: 144.4 [2C]: 169.7 [2C].

Elemental analysis calculated for C_50_H_86_N_4_O_6_S_2_ (Mmol = 903.38 g mol^-1^) (%): C = 66.48; H = 9.60; N = 6.20; found: C = 66.13; H = 9.25; N = 6.54.

tetramethylene-1,4-bis(decyldimethylammonium) diacesulfamate (**1b**)


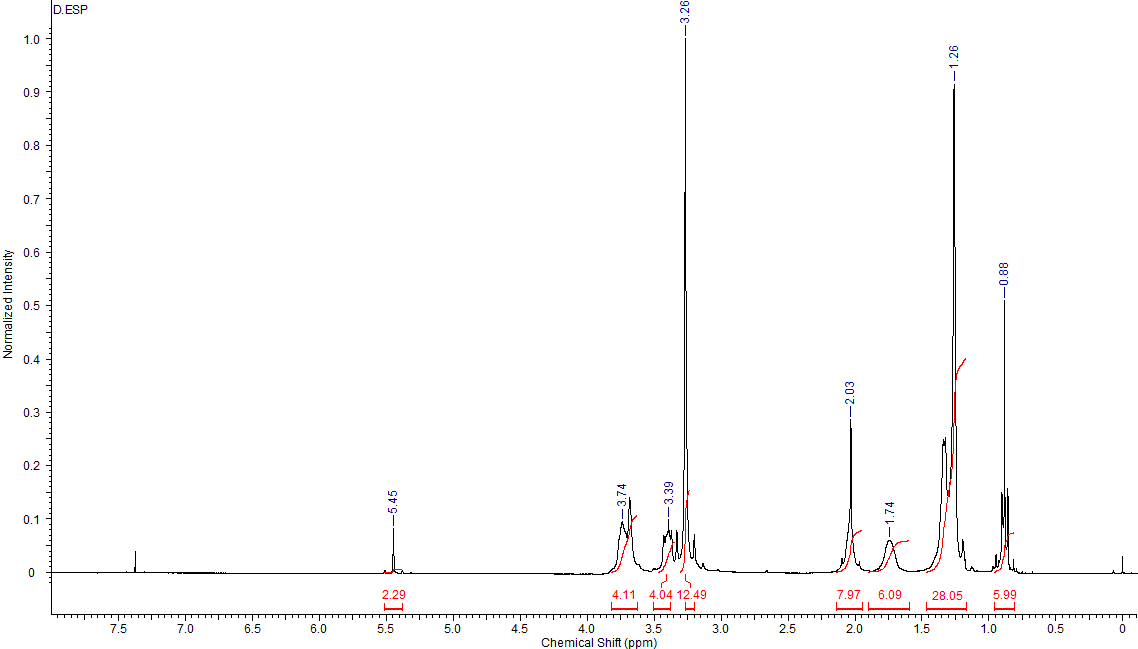


^1^H NMR (CDCl_3_) δ [ppm] = 0.88 (m. *J*=6.91 Hz. 6H): 1.26 (m. 28H): 1.74 (s. 6H): 2.03 (s. 8H): 3.26 (m. 12H): 3.39 (m. 4H): 3.74 (m. 4H): 5.45 (s. 2H):


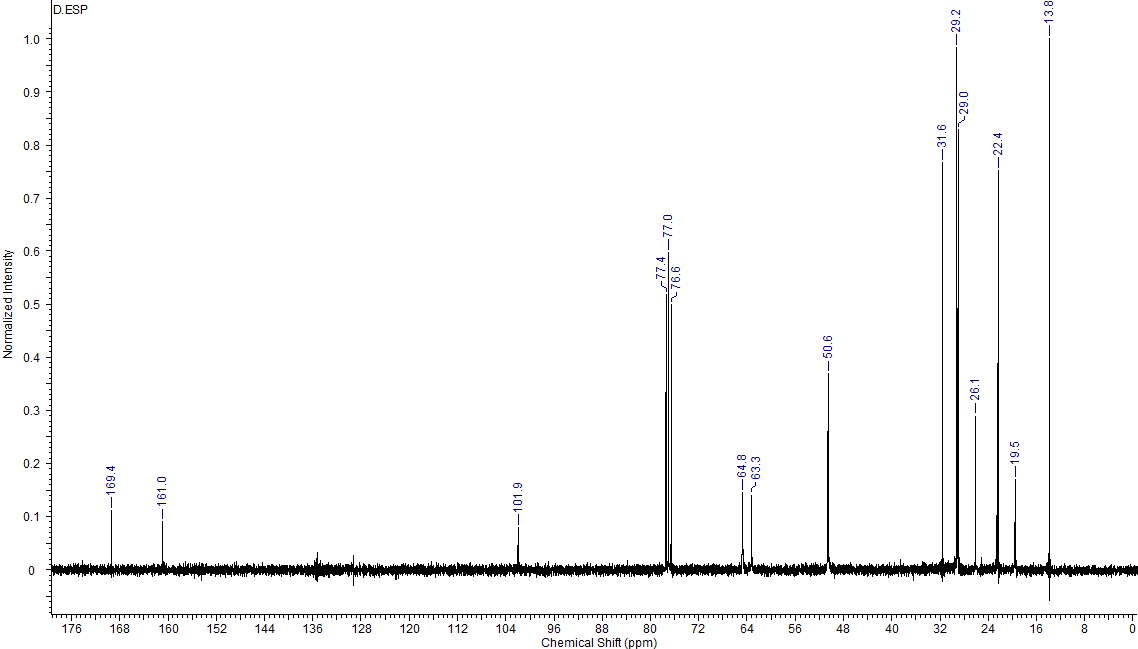


^13^C NMR (CDCl_3_) δ [ppm] = 13.8 [2C]: 19.5 [4C]: 22.4 [2C]: 26.1 [4C]: 29.0 [4C]: 29.2 [4C]: 31.6 [2C]: 50.6 [4C]: 63.3 [2C]: 64.8 [2C]: 101.9 [2C]: 161.0 [2C]: 169.4 [2C].

Elemental analysis calculated for C_36_H_70_N_4_O_8_S_2_ (Mmol = 751.10 g mol^-1^) (%): C = 57.57; H = 9.39; N = 7.46; found: C = 57.19; H = 9.02; N = 7.11.

hexamethylene-1,6-bis(decyldimethylammonium) diacesulfamate (**2b**)

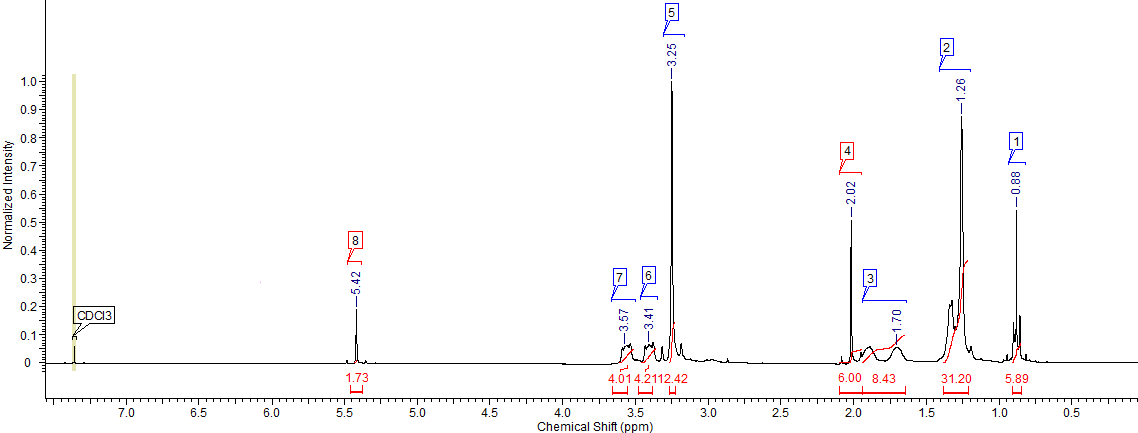


^1^H NMR (CDCl_3_) δ [ppm] = 0.88 (m. *J*=6.82 Hz. 6H): 1.26 (m. 32H): 1.70 (m. 8H): 2.02 (s. 6H): 3.25 (s. 12H): 3.41 (s. 4H): 3.57 (s. 4H): 5.42 (s. 2H):

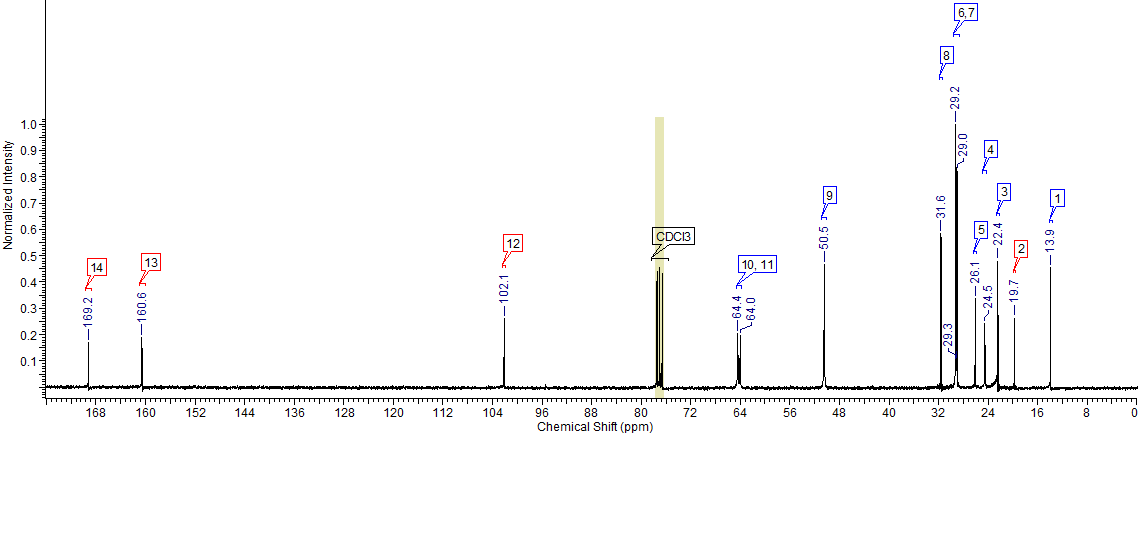


^13^C NMR (CDCl_3_) δ [ppm] = 13.9 [2C]: 19.7 [2C]: 22.4 [2C]: 24.5 [4C]: 26.1 [4C]: 29.0 [4C]: 29.2 [4C]: 31.6 [2C]: 50.5 [4C]: 64.0 [2C]: 64.4 [2C]: 102.1 [2C]: 160.6 [2C]: 169.2 [2C].

Elemental analysis calculated for C_38_H_74_N_4_O_8_S_2_ (Mmol = 779.15 g mol^-1^) (%): C = 58.58; H = 9.57; N = 7.19; found: C = 58.23; H = 9.21; N = 7.51.

octamethylene-1,8-bis(decyldimethylammonium) diacesulfamate (**3b**)


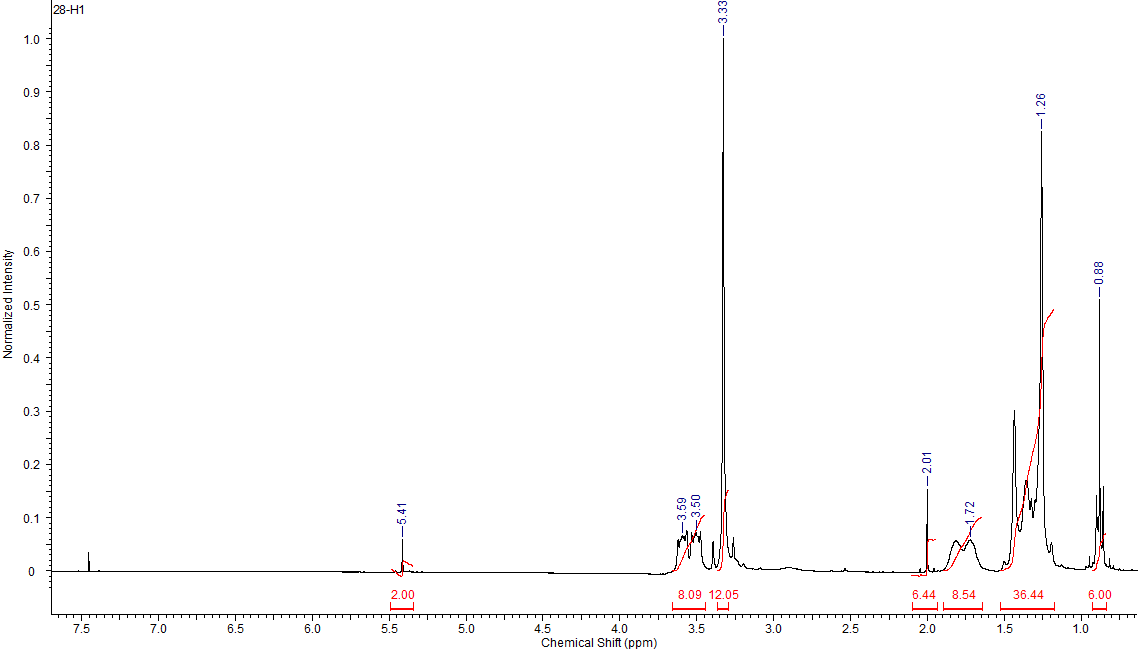


^1^H NMR (CDCl_3_) δ [ppm] = 0.88 (m. *J*=6.77 Hz. 6H): 1.26 (m. 36H): 1.72 (m. 8H): 2.01 (s. 6H): 3.33 (s. 12H): 3.50 (m. 8H): 5.41 (s. 2H):


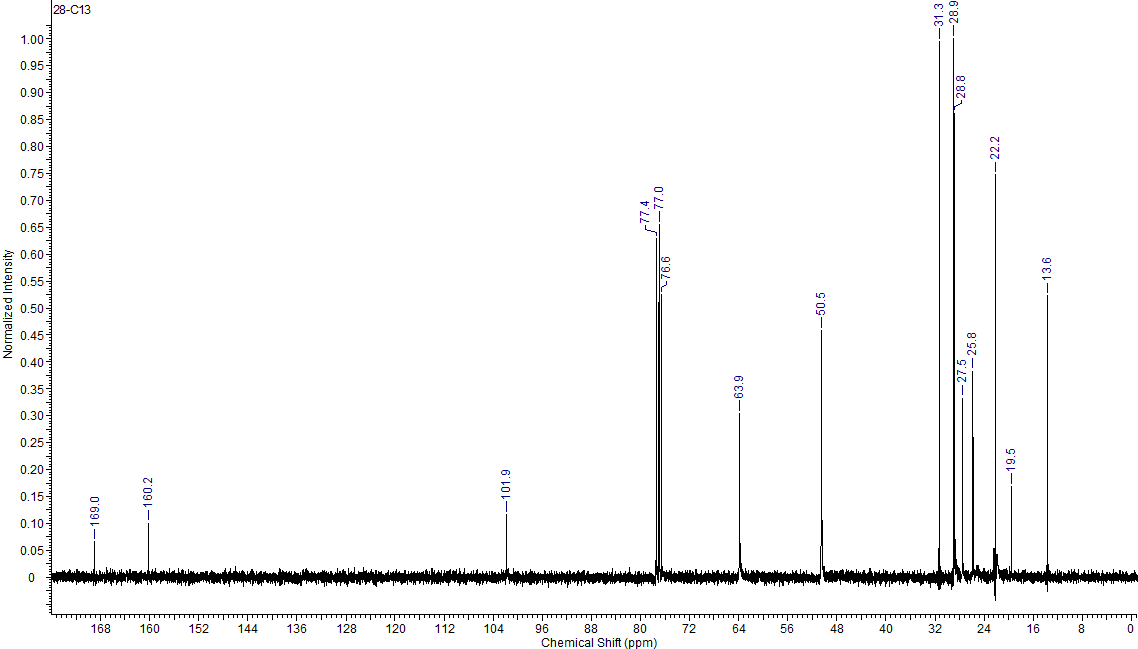


^13^C NMR (CDCl_3_) δ [ppm] = 13.6 [2C]: 19.5 [2C]: 22.2 [2C]: 25.8 [4C]: 27.5 [4C]: 28.8 [6C]: 28.9 [4C]: 31.3 [2C]: 50.5 [4C]: 63.9 [4C]: 101.9 [2C]: 160.2 [2C]: 169.0 [2C].

Elemental analysis calculated for C_40_H_78_N_4_O_8_S_2_ (Mmol = 807.20 g mol^-1^) (%): C = 59.52; H = 9.74; N = 6.94; found: C = 59.15; H = 9.38; N = 6.65.

decamethylene-1,10-bis(decyldimethylammonium) diacesulfamate (**4b**)


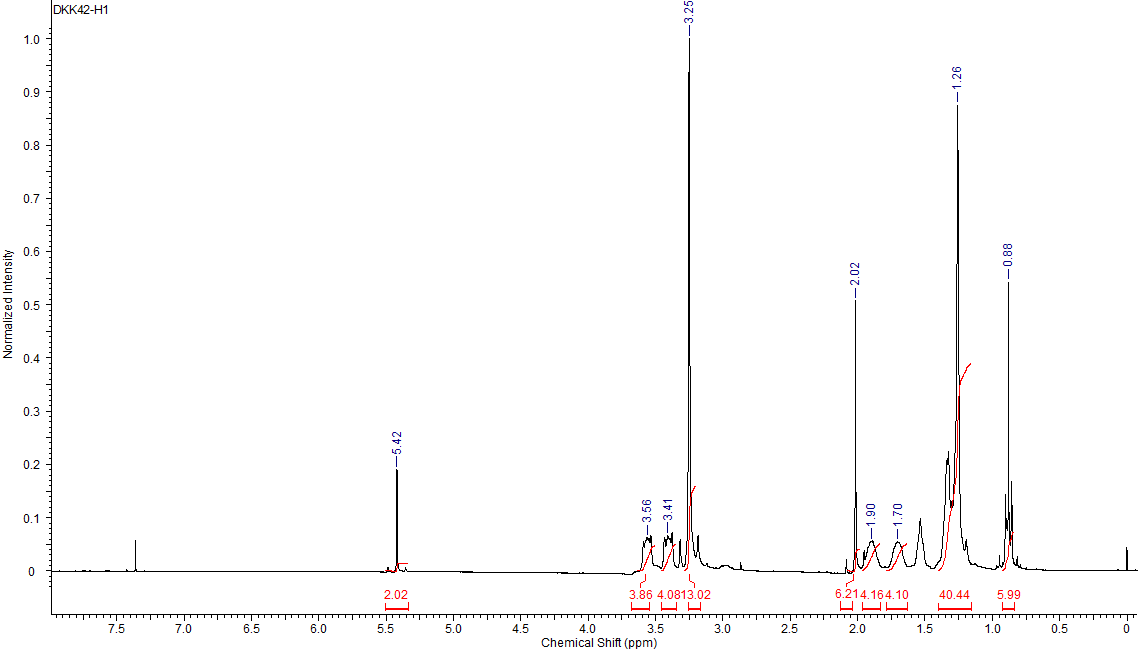


^1^H NMR (CDCl_3_) [ppm] = 0.88 (m. *J*=6.97 6H): 1.26 (m. 40H): 1.72 (s. 4H): 1.82 (s. 4H): 2.02 (s. 6H): 3.25 (m. 12H): 3.41 (s. 4H): 3.56 (s. 4H): 5.42 (s. 2H):


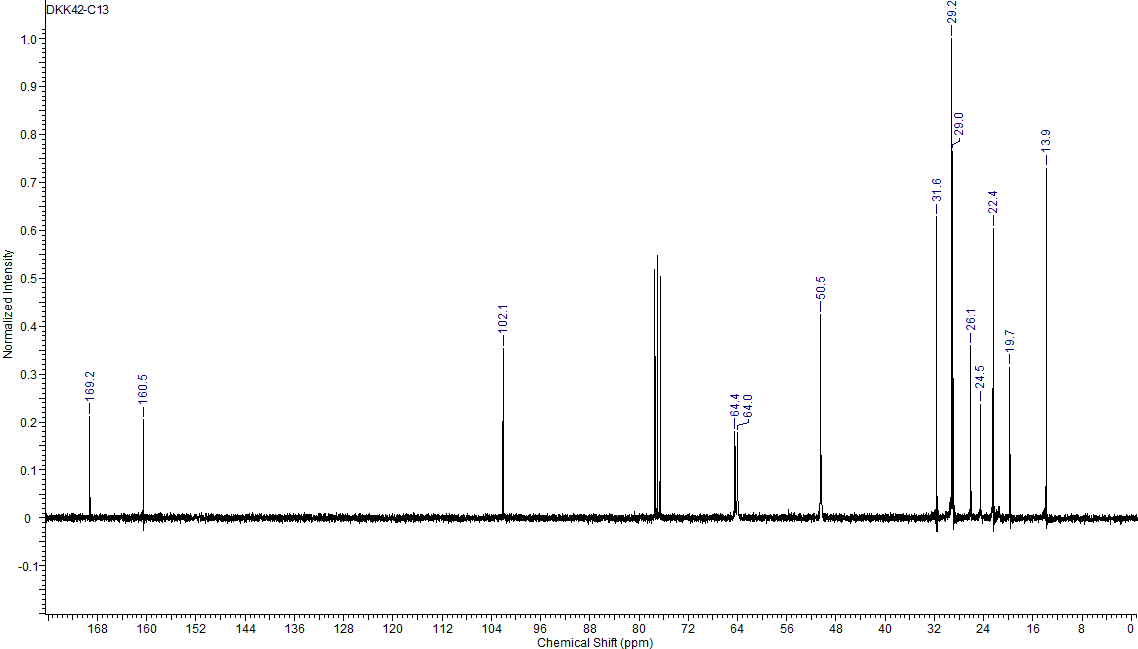


^13^C NMR (CDCl_3_) δ [ppm] = 13.9 [2C]: 19.7 [2C]: 22.4 [4C]: 24.5 [4C]: 26.1 [4C]: 29.0 [6C]: 29.2 [4C]: 31.6 [2C]: 50.5 [4C]: 64.0 [2C]: 64.4 [2C]: 102.1 [2C]: 160.5 [2C]: 169.2 [2C].

Elemental analysis calculated for C_42_H_82_N_4_O_8_S_2_ (Mmol = 835.26 g mol^-1^) (%): C = 60.40; H = 9.90; N = 6.71; found: C = 60.03; H = 9.52 N = 6.31.

dodecamethylene-1,12-bis(decyldimethylammonium) diacesulfamate (**5b**)


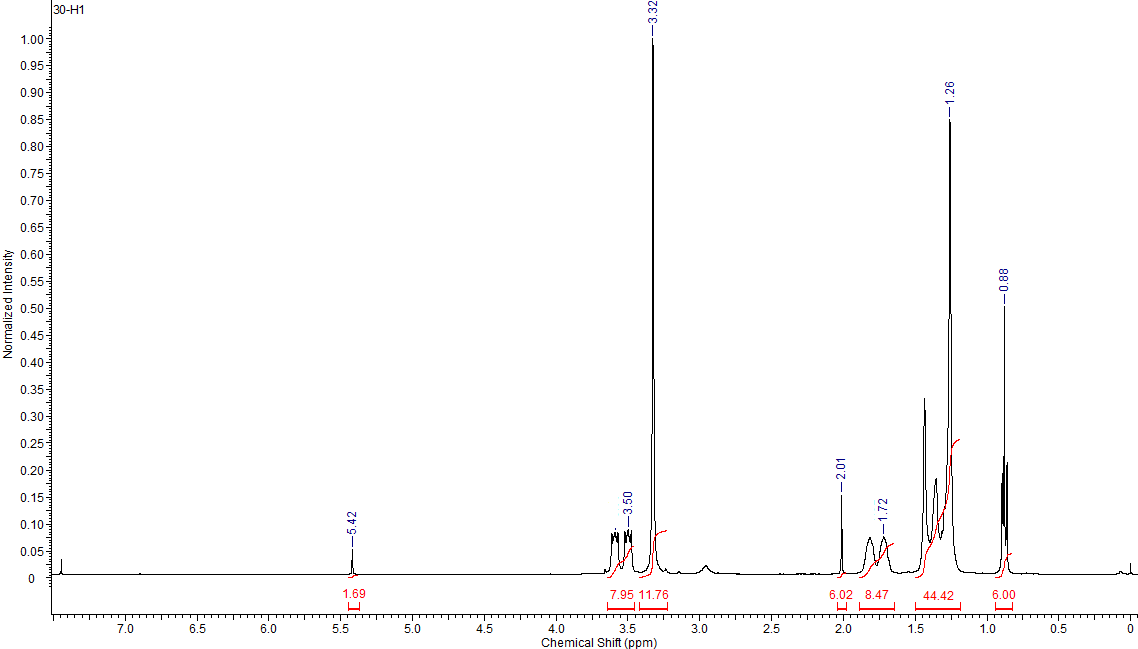


^1^H NMR (CDCl_3_) δ [ppm] = 0.88 (m. *J*=7.07 Hz. 6H): 1.26 (m. 44H): 1.72 (m. 8H): 2.01 (s. 6H): 3.32 (s. 12H): 3.50 (m. 8H): 5.44 (s. 2H):


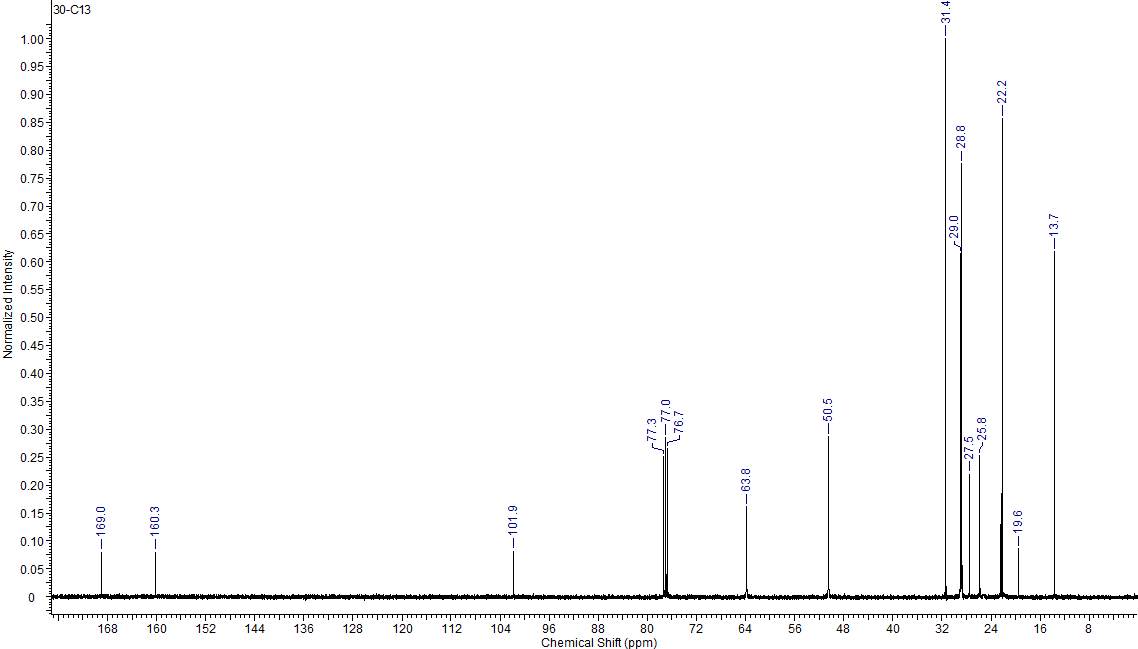


^13^C NMR (CDCl_3_) δ [ppm] = 13.7 [2C]: 19.6 [2C]: 22.2 [4C]: 25.8 [4C]: 27.5 [4C]: 28.8 [6C]: 29.0 [6C]: 31.4 [2C]: 50.5 [4C]: 63.8 [4C]: 101.9 [2C]: 160.3 [2C]: 169.0 [2C].

Elemental analysis calculated for C_44_H_86_N_4_O_8_S_2_ (Mmol = 863.31 g mol^-1^) (%): C = 61.22; H = 10.04; N = 6.49; found: C = 61.57; H = 10.38; N = 6.07.

tetramethylene-1,4-bis(decyldimethylammonium) dilactate (**1c**)


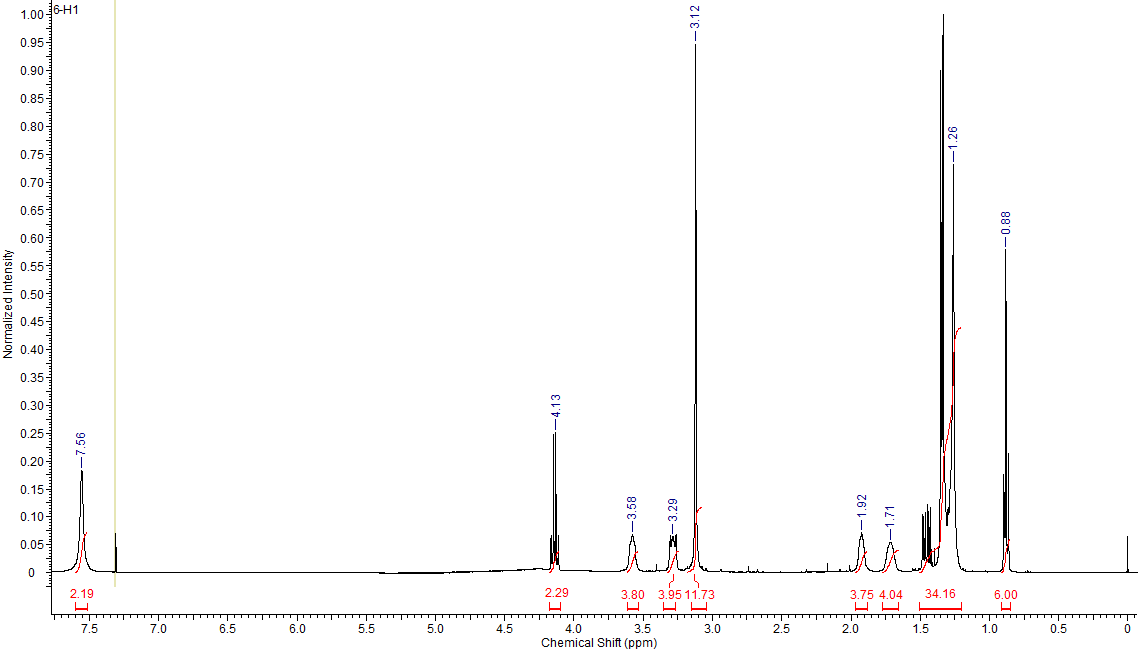


^1^H NMR (CDCl_3_) δ [ppm] = 0.88 (m. *J*=6.7 Hz. 6H): 1.26 (m. 34H): 1.71 (s. 4H): 1.92 (s. 4H): 3.12 (s. 12H): 3.29 (s. 4H): 3.58 (s. 4H): 4.13 (m. *J*= 6.9 Hz 2H): 7.56 (s. 2H):


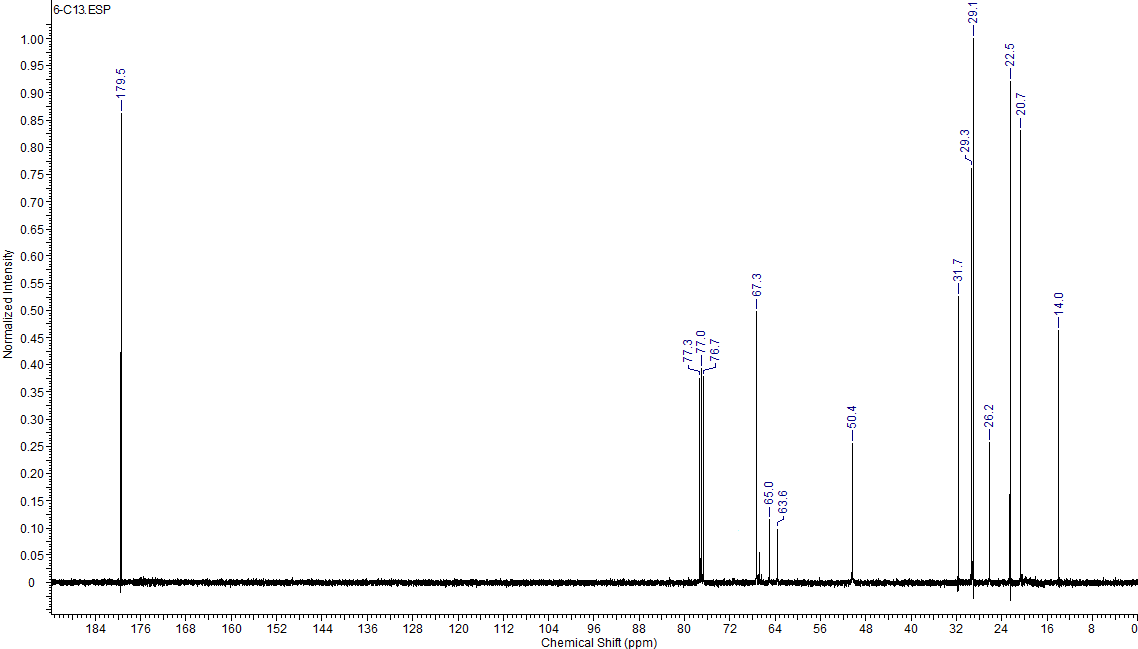


^13^C NMR (CDCl_3_) δ [ppm] = 14.0 [2C]: 20.7 [4C]: 22.5 [2C]: 26.2 [4C]: 29.1 [4C]: 29.3 [4C]: 31.7 [2C]: 50.4 [4C]: 63.6 [2C]: 65.0 [2C]: 67.3 [2C]: 179.5 [2C].

Elemental analysis calculated for C_34_H_72_N_2_O_6_ (Mmol = 604.96 g mol^-1^) (%): C = 67.50; H = 12.00; N = 4.63; found: C = 67.15; H = 12.31; N = 4.25.

hexamethylene-1.6-bis(decyldimethylammonium) dilactate (**2c**)


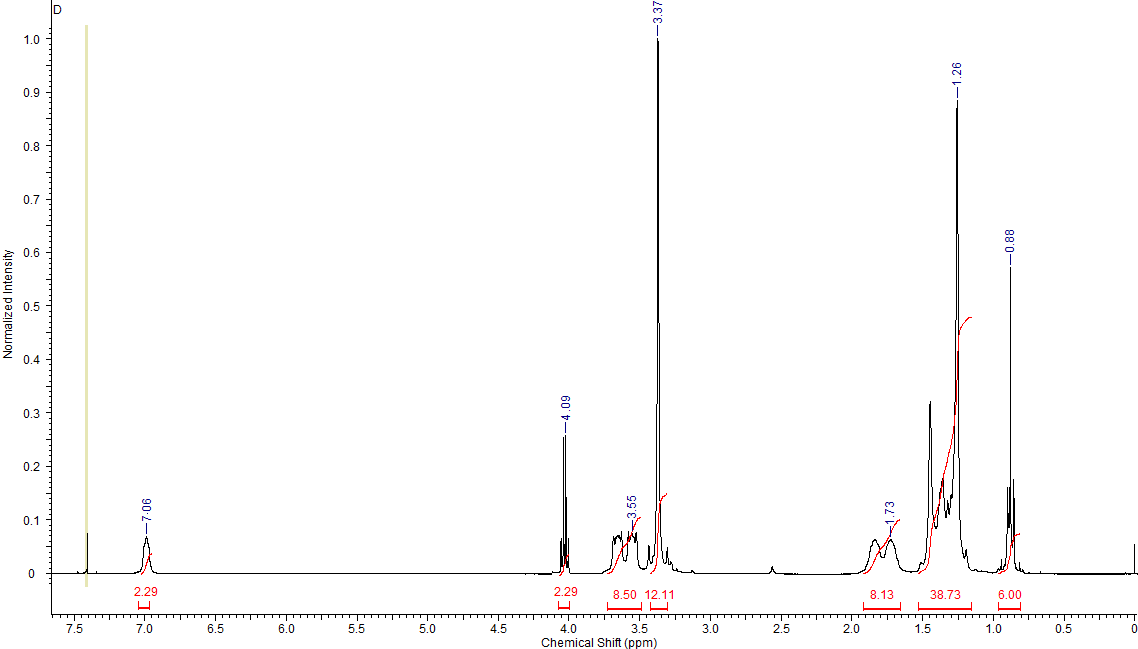


^1^H NMR (CDCl_3_) δ [ppm] = 0.88 (m. *J*=7.0 Hz. 6H): 1.26 (m. 38H): 1.73 (m. 8H): 3.37 (s. 12H): 3.55 (m. 8H): 4.09 (m. *J*= 6.9 Hz 2H): 7.06 (s. 2H):


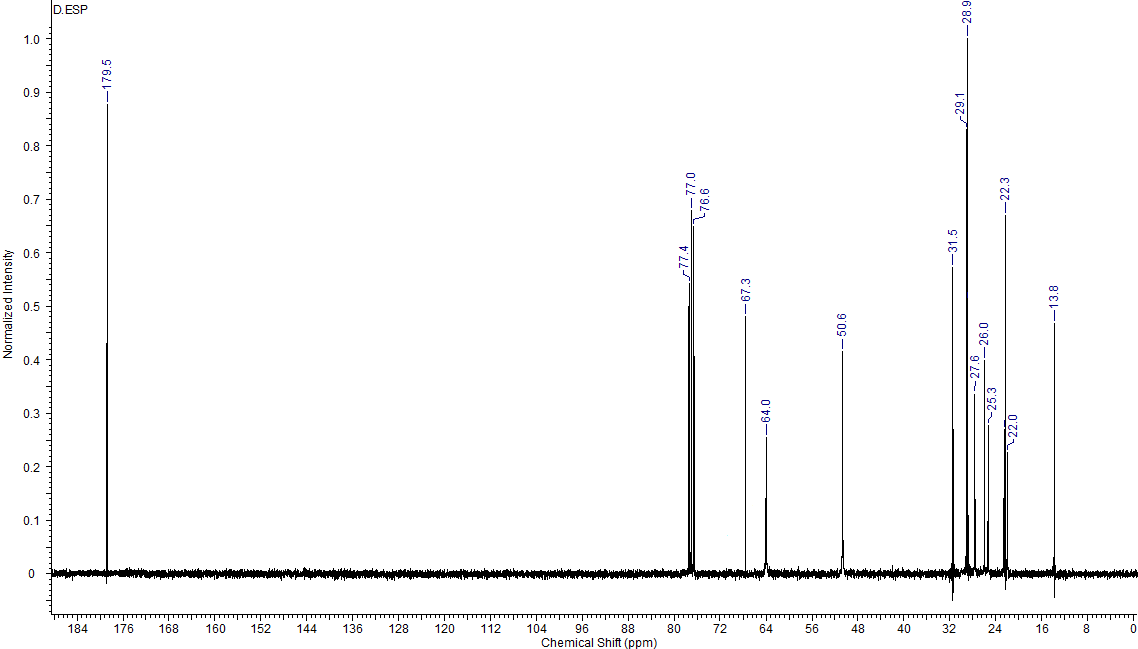


^13^C NMR (CDCl_3_) δ [ppm] = 13.8 [2C]: 22.0 [2C]: 22.3 [2C]: 25.3 [4C]: 26.0 [4C]: 28.9 [4C]: 29.1 [4C]: 31.5 [2C]: 50.6 [4C]: 64.0 [4C]: 67.4 [2C]: 179.5 [2C].

Elemental analysis calculated for C_36_H_76_N_2_O_6_ (Mmol = 633.01 g mol^-1^) (%): C = 68.31; H = 12.10; N = 4.43; found: C = 68.00; H = 12.41; N = 4.11.

octamethylene-1,8-bis(decyldimethylammonium) dilactate (**3c**)

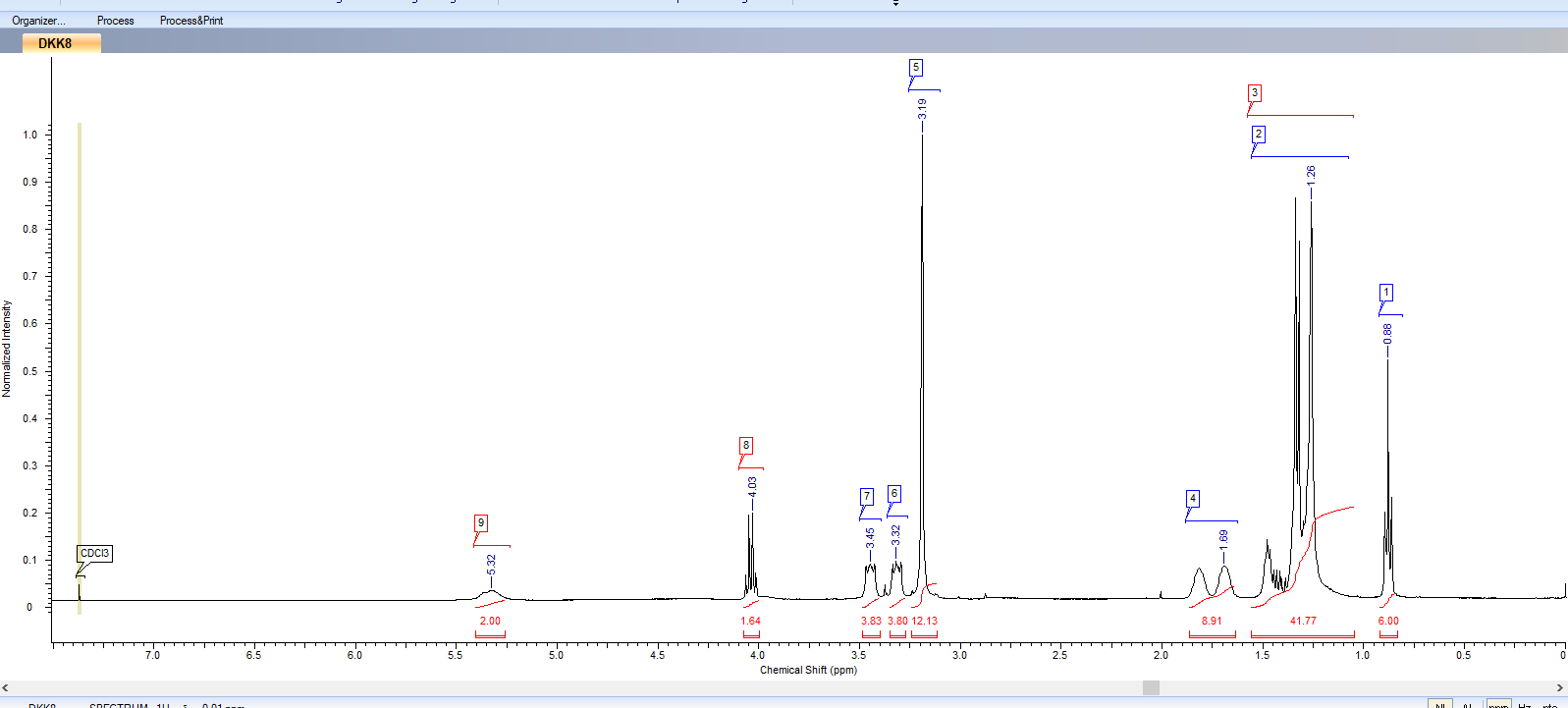


^1^H NMR (CDCl_3_) δ [ppm] = 0.88 (m. *J*=6.9 Hz. 6H): 1.26 (m. 42H): 1.69 (m. 8H): 3.19 (s. 12H): 3.32 (s. 4H): 3.45 (s. 4H): 4.03 (m. *J*=6.9 Hz. 2H): 5.32 (s. 2H):

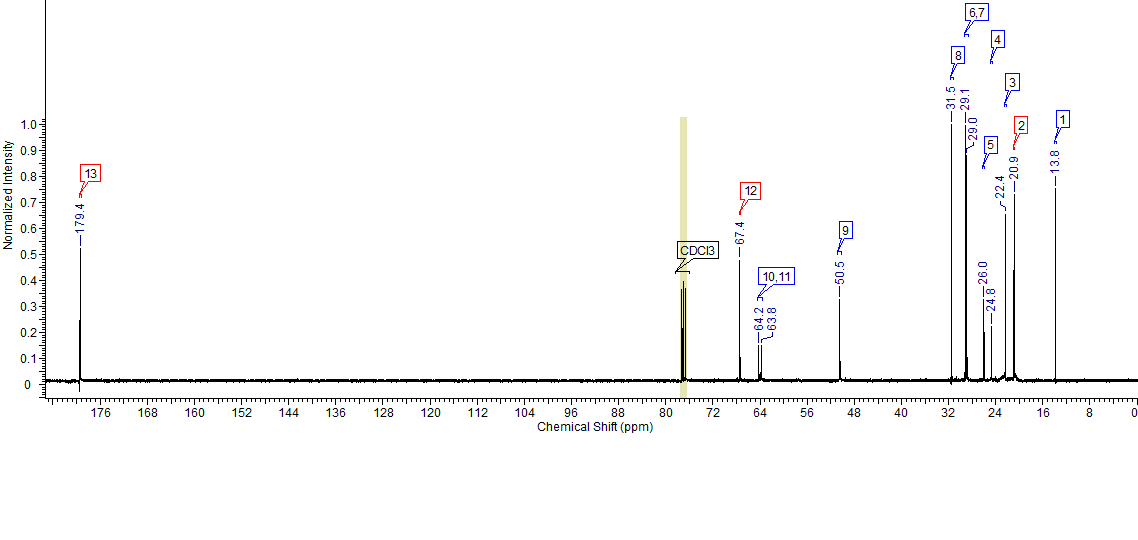


^13^C NMR (CDCl_3_) δ [ppm] = 13.8 [2C]: 20.9 [2C]: 22.4 [2C]: 24.8 [4C] 26.0 [4C]: 29.0 [6C]: 29.1 [4C]: 31.5 [2C]: 50.5 [4C]: 63.8 [2C]: 64.2 [2C]: 67.4 [2C]: 179.4 [2C].

Elemental analysis calculated for C_38_H_80_N_2_O_6_ (Mmol = 661.07 g mol^-1^) (%): C = 69.04; H = 12.20; N = 4.24; found: C = 68.78; H = 11.81; N = 4.64.

decamethylene-1,10-bis(decyldimethylammonium) dilactate (**4c**)


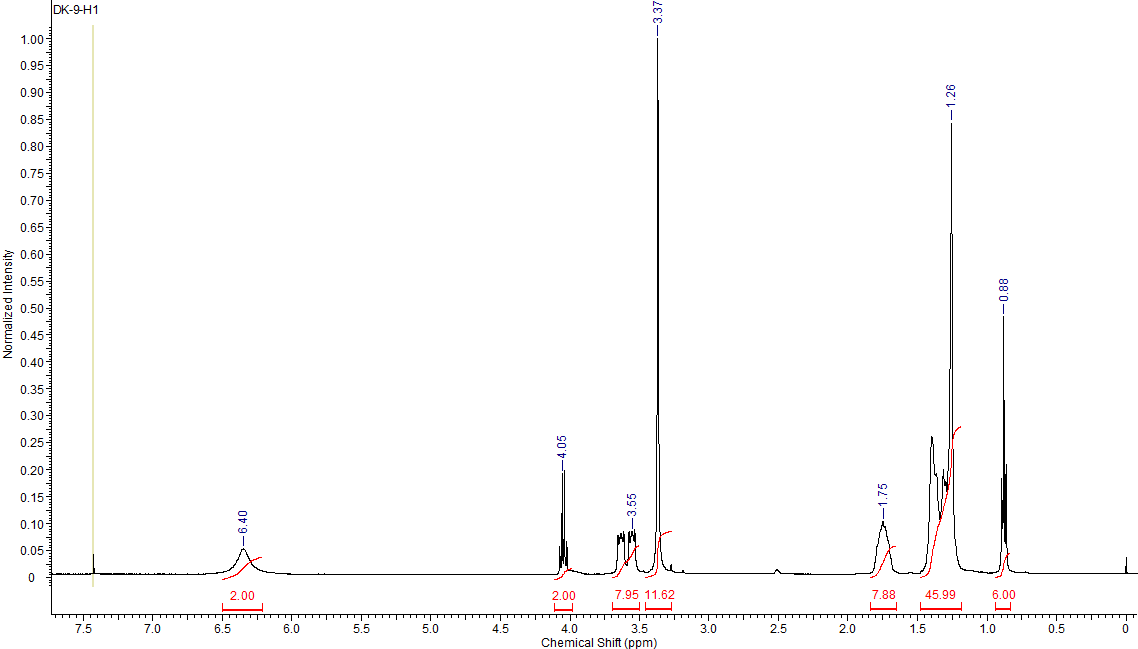


^1^H NMR (CDCl_3_) δ [ppm] = 0.88 (m. *J*=6.9 Hz. 6H): 1.26 (m. 46H): 1.75 (s. 8H): 3.37 (s. 12H): 3.55 (s. 8H): 4.05 (m. *J*=6.8 Hz. 2H): 6.40 (s. 2H):


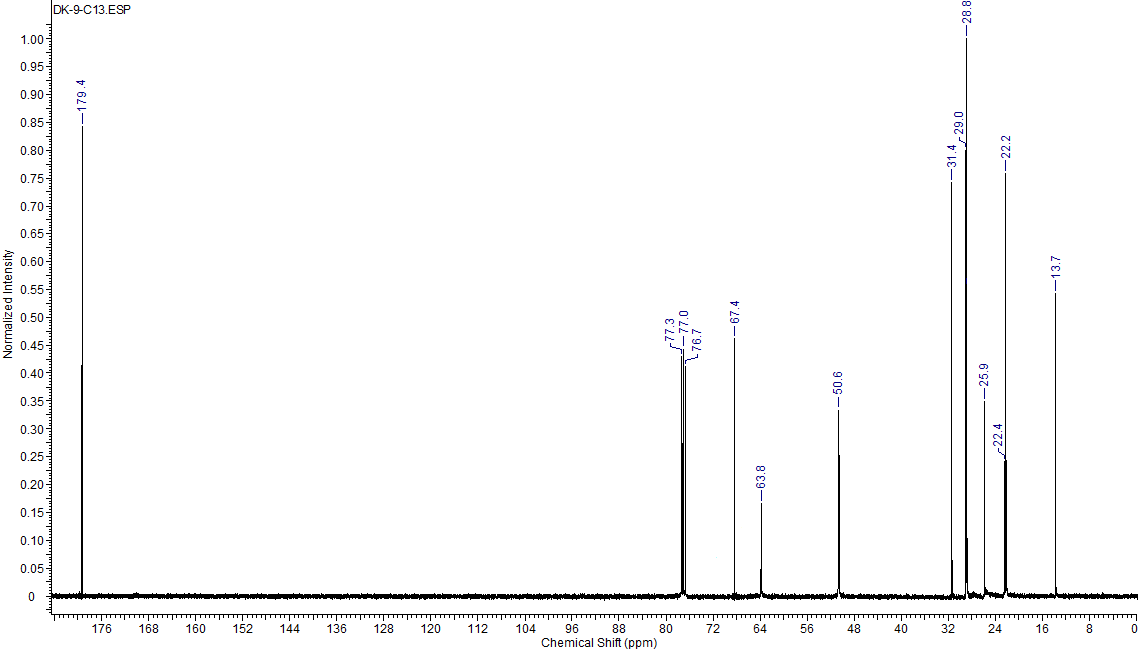


^13^C NMR (CDCl_3_) δ [ppm] = 13.7 [2C]: 22.2 [2C]: 22.4 [2C]: 25.9 [8C]: 28.8 [6C]: 29.0 [8C]: 31.4 [2C]: 50.6 [4C]: 63.8 [4C]: 67.4 [2C]: 179.4 [2C].

Elemental analysis calculated for C_40_H_84_N_2_O_6_ (Mmol = 689.12 g mol^-1^) (%): C = 69.72; H = 12.29; N = 4.07; found: C = 69.33; H = 11.98; N = 4.41.

dodecamethylene-1,12-bis(decyldimethylammonium) dilactate (**5c**)


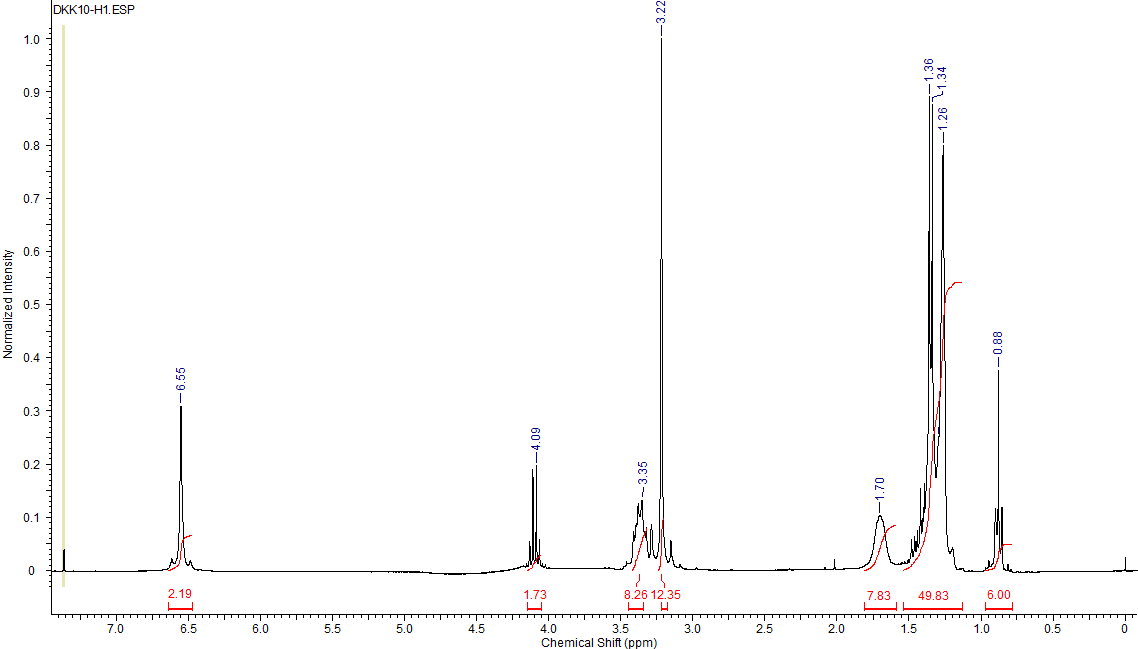


^1^H NMR (CDCl_3_) δ [ppm] = 0.88 (m. *J*=6.62 Hz. 6H): 1.26 (m. 50H): 1.70 (s. 8H): 3.22 (m. 12H): 3.35 (s. 8H): 4.09 (m. *J* = 6.56 Hz. 2H): 6.55 (s. 2H):


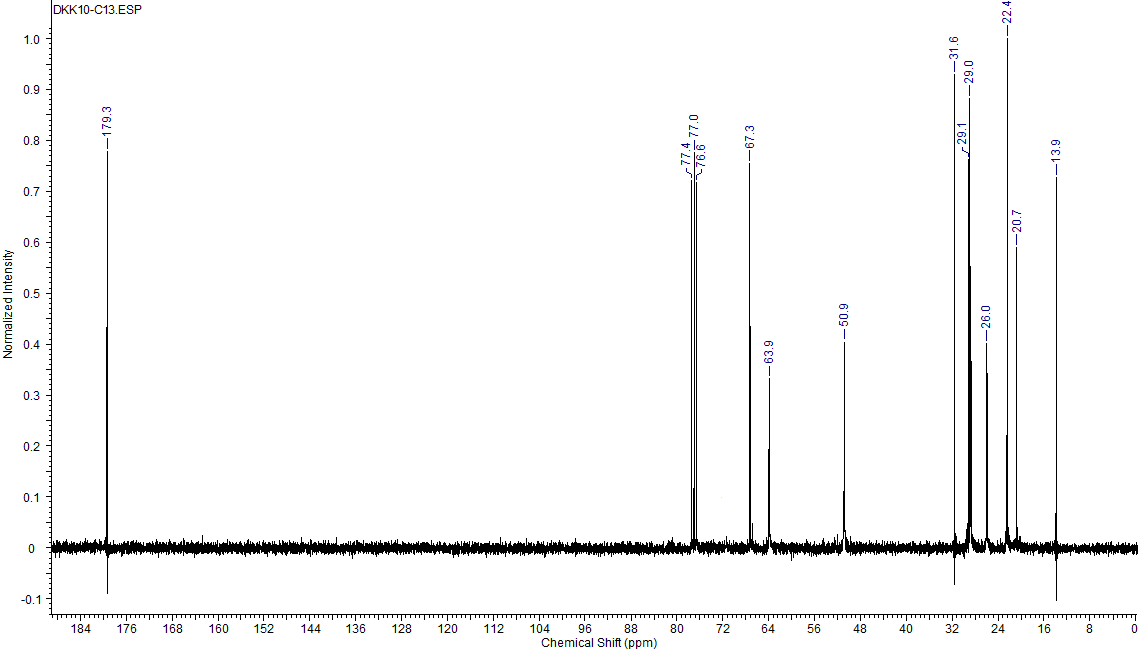


^13^C NMR (CDCl_3_) δ [ppm] = 13.9 [2C]: 20.7 [2C]: 22.4 [2C]: 26.0 [8C]: 29.0 [6C]: 29.1 [8C]: 31.6 [2C]: 50.9 [4C]: 63.9 [4C]: 67.3 [2C]: 179.3 [2C].

Elemental analysis calculated for C_42_H_88_N_2_O_6_ (Mmol = 717.17 g mol^-1^) (%): C = 70.34; H = 12.37; N = 3.91; found: C = 70.01; H = 12.77; N = 4.51.

tetramethylene-1,4-bis(decyldimethylammonium) dipiroglutamate (**1d**)


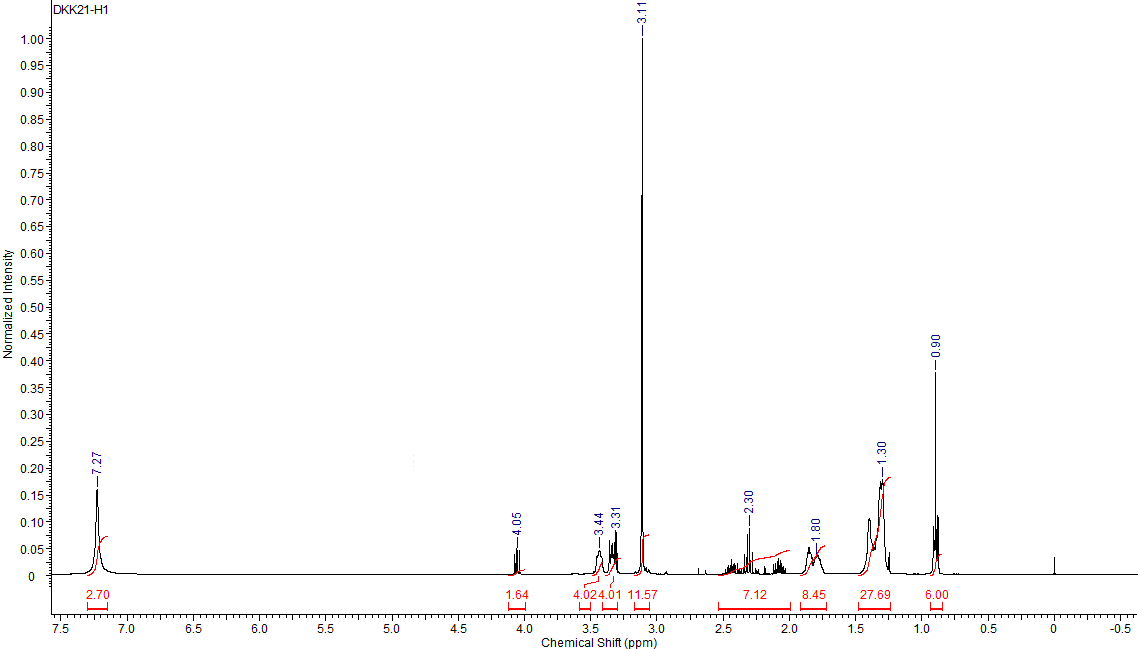


^1^H NMR (MeOH) δ [ppm] = 0.90 (m. *J*=7.28 Hz. 6H): 1.30 (m. 28H): 1.80 (m. 8H): 2.30 (m. 8H): 3.11 (s. 12H): 3.31 (s. 4H): 3.44 (s. 4H): 4.05 (m. 2H): 7.27 (s. 2H):

^13^C NMR (CDCl_3_) δ [ppm] = 14.5 [2C]: 20.7 [2C]: 23.7 [2C]: 23.8 [2C]: 27.1 [4C]: 27.5 [4C]: 30.3 [2C]: 30.6 [2C]: 31.2 [2C]: 33.1 [2C]: 51.4 [4C]: 59.8 [2C]: 64.3 [2C]: 65.8 [2C]: 179.7 [2C]: 181.0 [2C].

Elemental analysis calculated for C_38_H_74_N_4_O_6_ (Mmol = 683.03 g mol^-1^) (%): C = 66.82; H = 10.92; N = 8.20; found: C = 66.45; H = 10.52; N = 8.51.

hexamethylene-1,6-bis(decyldimethylammonium) dipiroglutamate (**2d**)


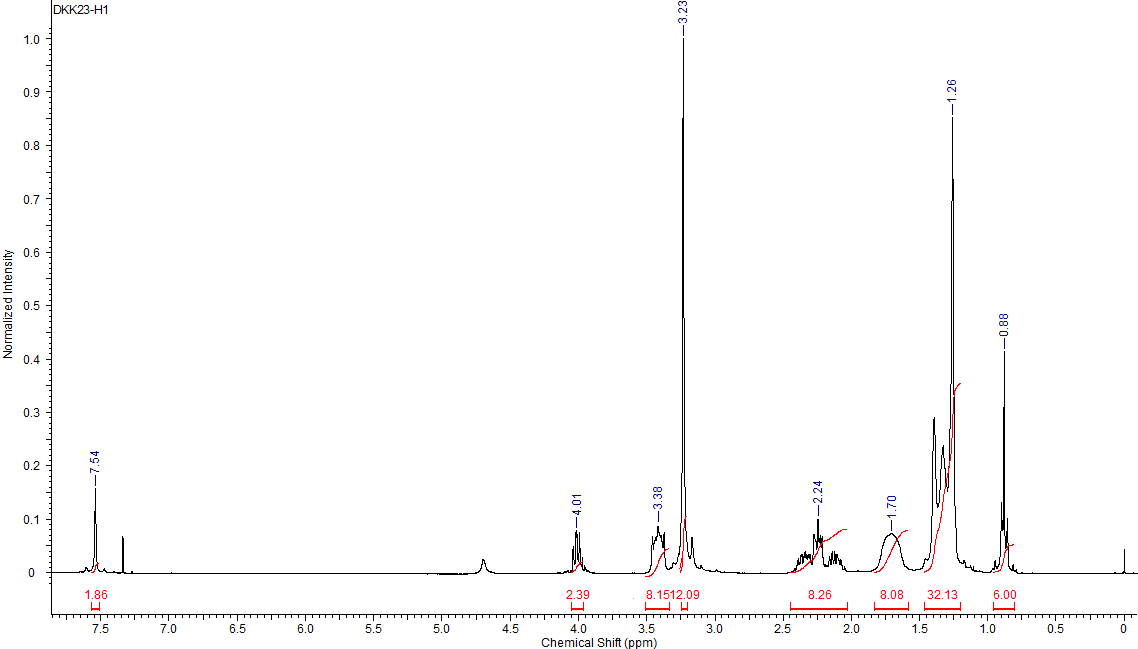


^1^H NMR (CDCl_3_) δ [ppm] = 0.88 (m. *J*=7.01 Hz. 6H): 1.26 (m. 32H): 1.70 (s. 8H): 2.24 (m. 8H): 3.23 (s. 12H): 3.35 (s. 8H): 4.01 (m. 2H): 7.54 (s. 2H):


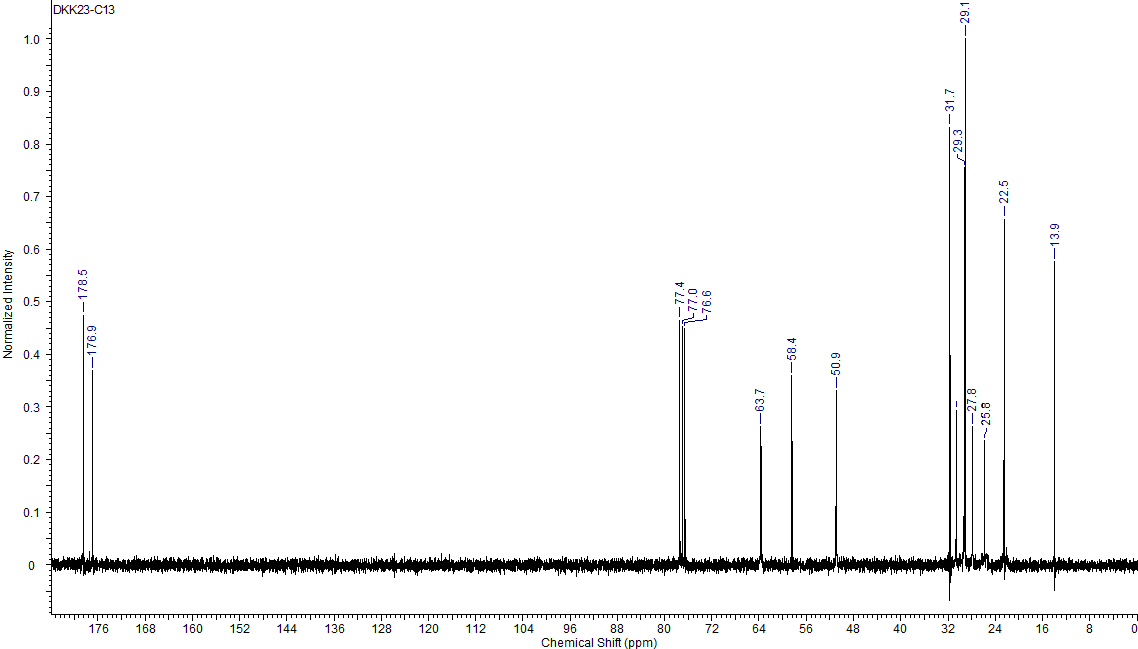


^13^C NMR (CDCl_3_) δ [ppm] = 13.9 [2C]: 22.5 [2C]: 26.8 [4C]: 27.8 [6C]: 29.1 [4C]: 29.3 [4C]: 30.6 [2C]: 31.7 [2C]: 50.9 [4C]: 58.4 [2C]: 63.7 [4C]: 176.9 [2C]: 178.5 [2C].

Elemental analysis calculated for C_40_H_78_N_4_O_6_ (Mmol = 711.09 g mol^-1^) (%): C = 67.56; H = 11.06; N = 7.88; found: C = 67.22; H = 11.40; N = 7.42.

octamethylene-1.8-bis(decyldimethylammonium) dipiroglutamate (**3d**)


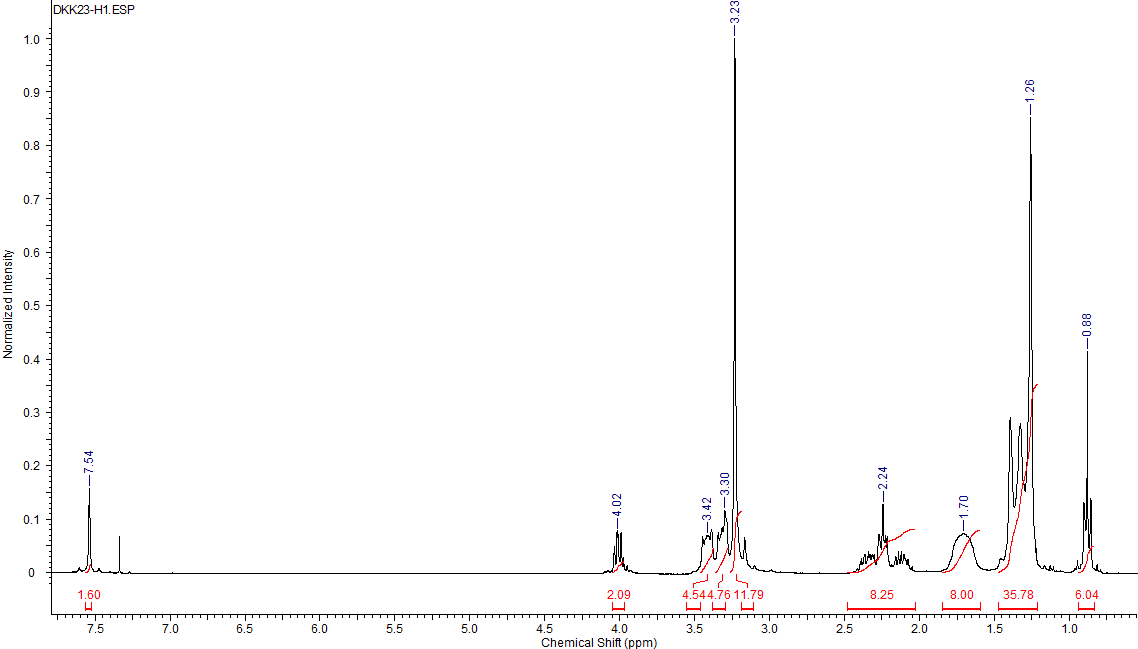


^1^H NMR (CDCl_3_) δ [ppm] = 0.88 (m. *J*=6.93 Hz. 6H): 1.26 (m. 36H): 1.70 (s. 8H): 2.24 (m. 8H): 3.23 (s. 12H): 3.30 (s. 4H): 3.42 (s. 4H): 4.02 (m. 2H): 7.54 (s. 2H):


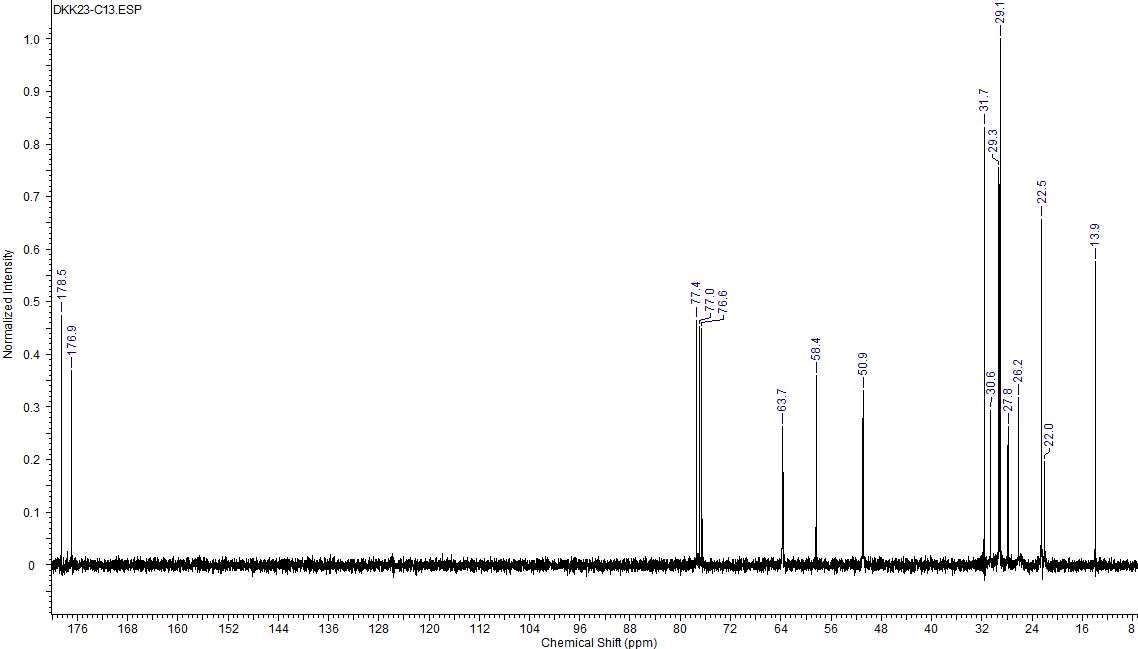


^13^C NMR (CDCl_3_) δ [ppm] = 13.9 [2C]: 22.0 [4C]: 22.5 [2C]: 26.2 [4C]: 27.8 [6C]: 29.1 [4C]: 29.3 [4C]: 30.6 [2C]: 31.7 [2C]: 50.9 [4C]: 58.4 [2C]: 63.7 [4C]: 176.9 [2C]: 178.5 [2C].

Elemental analysis calculated for C_42_H_82_N_4_O_6_ (Mmol = 739.14 g mol^-1^) (%): C = 68.25; H = 11.18; N = 7.58; found: C = 68.62 H = 10.83 N = 7.14.

decamethylene-1,10-bis(decyldimethylammonium) dipiroglutamate (**4d**)


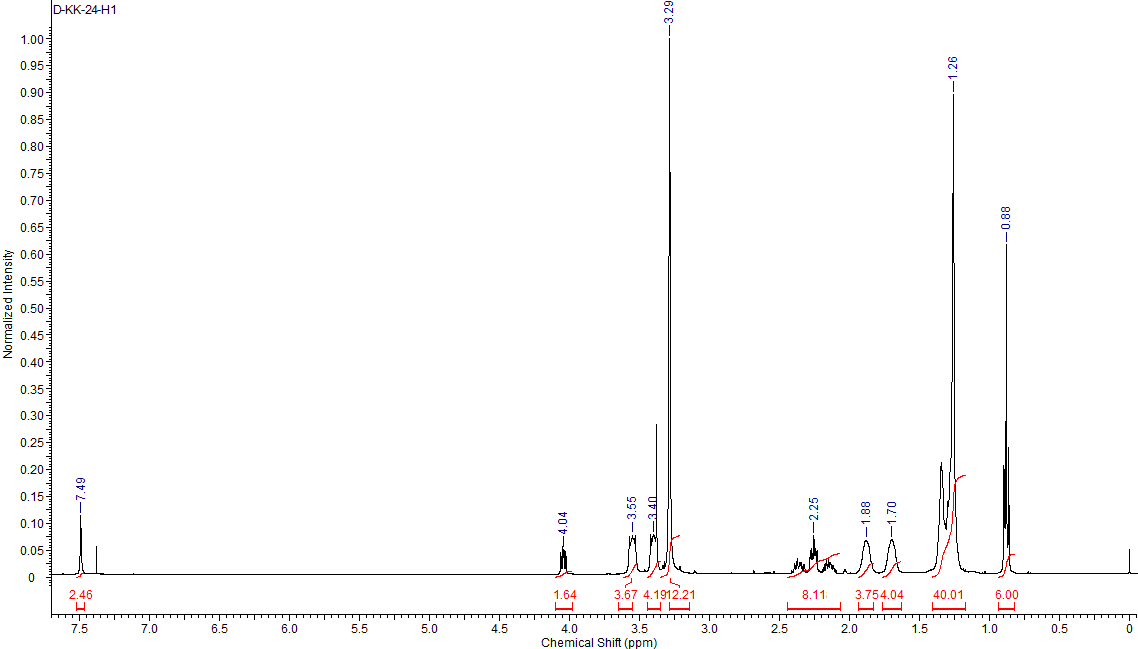


^1^H NMR (CDCl_3_) δ [ppm] = 0.88 (m. *J*=6.98 Hz. 6H): 1.26 (m. 40H): 1.70 (s. 4H): 1.88 (s. 4H): 2.26 (m. 8H): 3.29 (s. 12H): 3.40 (s. 4H): 3.55 (s. 4H): 4.03 (m. 2H): 7.49 (s. 2H):


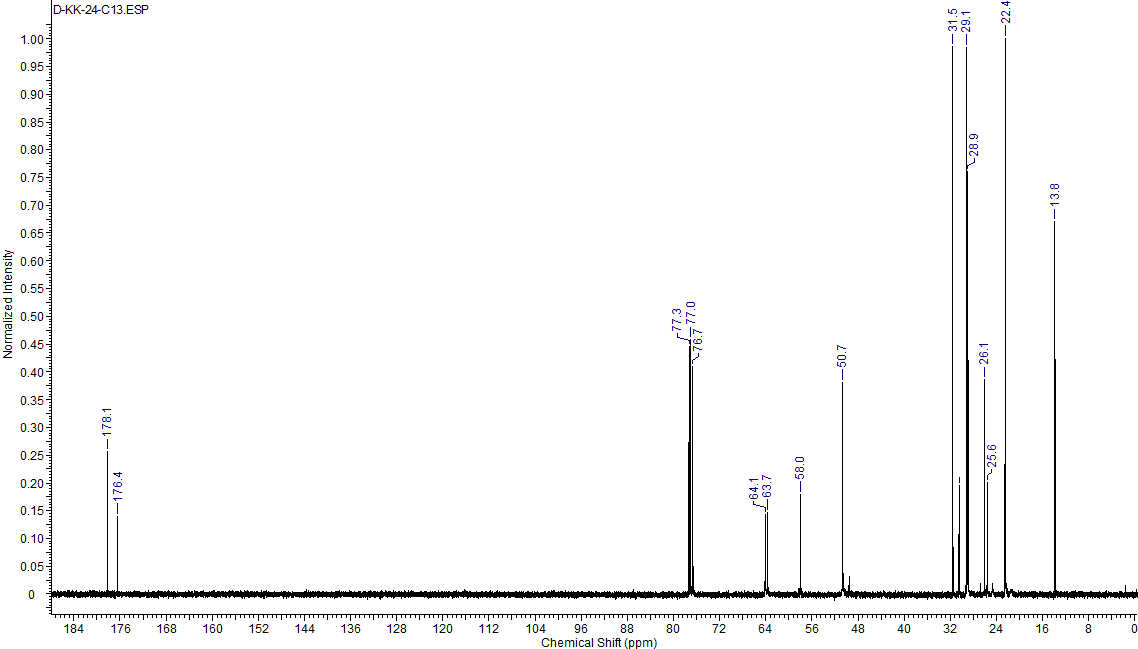


^13^C NMR (CDCl_3_) δ [ppm] = 13.8 [2C]: 22.4 [2C]: 25.6 [4C]: 26.1 [6C]: 28.9 [6C]: 29.1 [6C]: 30.5 [2C]: 31.5 [2C]: 50.7 [4C]: 58.0 [2C]: 63.7 [2C]: 64.1 [2C]: 176.4 [2C]: 178.1 [2C].

Elemental analysis calculated for C_44_H_86_N_4_O_6_ (Mmol = 767.19 g mol^-1^) (%): C = 68.89; H = 11.30; N = 7.30; found: C = 68.49 H = 11.62 N =7.64.

dodecamethylene-1,12-bis(decyldimethylammonium) dipiroglutamate (**5d**)

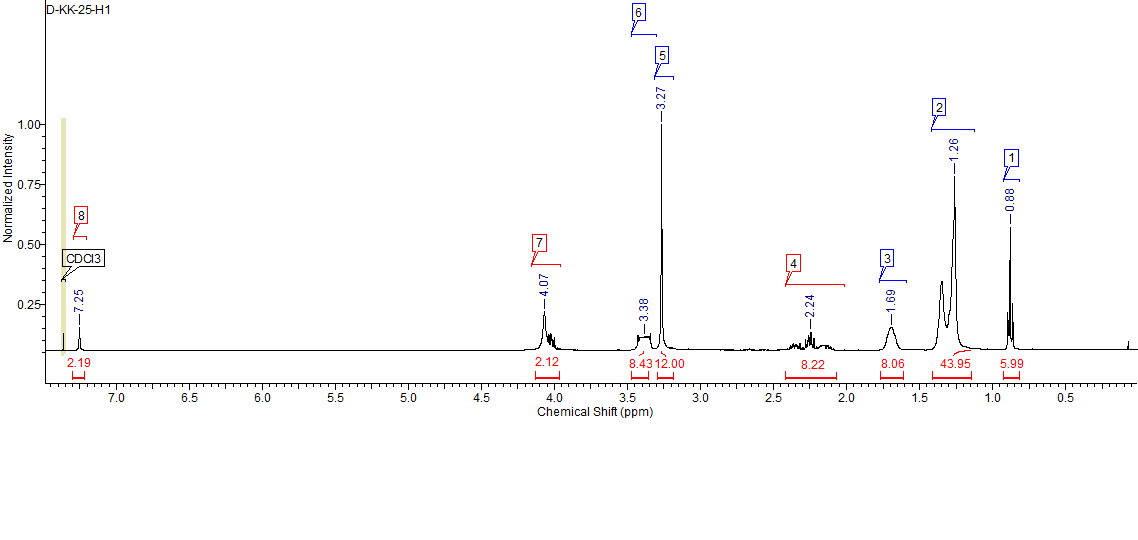


^1^H NMR (CDCl_3_) δ [ppm] = 0.88 (m. *J* = 7.14 Hz. 6H): 1.26 (m. 44H): 1.69 (m. 8H): 2.24 (m. 8H): 3.27 (m. 12H): 3.38 (s. 8H): 4.07 (m. 2H): 7.25 (s. 2H):

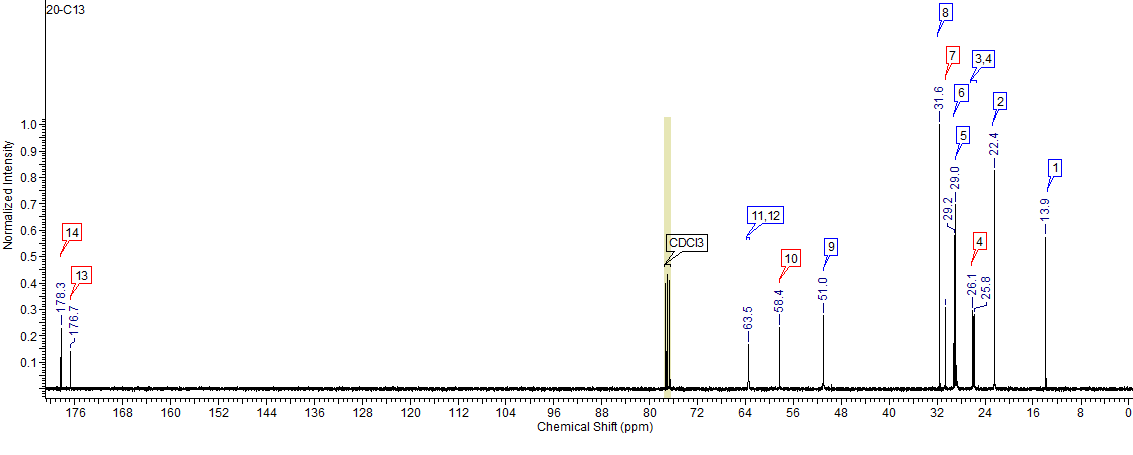


^13^C NMR (CDCl_3_) δ [ppm] = 13.9 [2C]: 22.4 [2C]: 25.8 [4C]: 26.1 [6C]; 29.0 [6C]: 29.2 [8C]: 30.6 [2C]: 31.6 [2C]: 51.0 [4C]: 58.4 [2C]: 63.5 [2C]: 63.5 [2C]: 176.7 [2C]: 178.3 [2C].

Elemental analysis calculated for C_46_H_90_N_4_O_6_ (Mmol = 795.25 g mol^-1^) (%): C = 69.48; H = 11.41; N = 7.05; found: C = 69.13; H = 10.11; N = 7.41.

| **Compound** | **Granary weevil** | | | | | | **Confused ﬂour beetle** | | | | | | | | | | | | **Khapra beetle** | | | | | |
| --- | --- | --- | --- | --- | --- | --- | --- | --- | --- | --- | --- | --- | --- | --- | --- | --- | --- | --- | --- | --- | --- | --- | --- | --- |
|  | (*Sitophilus granarius*) | | | | | | (*Tribolium confusum*) | | | | | | | | | | | | (*Trogoderma granarium*) | | | | | |
|  | Adults | | | | | | Adults | | | | | | Larvae | | | | | | Larvae | | | | | |
|  | R^a^ | | A | | T | | R | | A | | T | | R | | A | | T | | R | | A | | T | |
| **1a** | 89 | bcd | 58 | abcd | 147 | bcd | 86 | b | 55 | ab | 141 | b | 100 | a | 93 | ab | 193 | ab | 97 | b | 99 | c | 196 | b |
| **2a** | 81 | ab | 53 | abc | 134 | abc | 94 | b | 64 | abc | 158 | bcd | 85 | a | 91 | ab | 175 | ab | 96 | b | 90 | bc | 186 | b |
| **3a** | 89 | bcd | 68 | abcde | 156 | bcde | 97 | b | 69 | abcd | 166 | bcde | 97 | a | 86 | a | 184 | ab | 99 | b | 99 | c | 199 | b |
| **4a** | 99 | cd | 77 | cde | 176 | def | 99 | b | 86 | bcd | 184 | de | 100 | a | 96 | ab | 196 | ab | 99 | b | 95 | c | 193 | b |
| **5a** | 100 | d | 87 | de | 187 | ef | 100 | b | 77 | abcd | 177 | bcde | 100 | a | 100 | b | 200 | b | 100 | b | 100 | c | 200 | b |
| **average** | 92 |  | 68 |  | 160 |  | 95 |  | 70 |  | 165 |  | 96 |  | 93 |  | 190 |  | 98 |  | 97 |  | 195 |  |
| **1b** | 99 | cd | 80 | cde | 179 | def | 96 | b | 65 | abc | 161 | bcd | 100 | a | 92 | ab | 192 | ab | 100 | b | 96 | c | 196 | b |
| **2b** | 88 | bcd | 59 | abcd | 147 | bcd | 99 | b | 74 | abcd | 173 | bcde | 97 | a | 96 | ab | 193 | ab | 99 | b | 93 | c | 192 | b |
| **3b** | 90 | bcd | 75 | bcde | 165 | cdef | 99 | b | 88 | bcd | 187 | de | 100 | a | 90 | ab | 190 | ab | 100 | b | 99 | c | 199 | b |
| **4b** | 100 | d | 76 | bcde | 176 | def | 100 | b | 88 | bcd | 188 | de | 98 | a | 94 | ab | 192 | ab | 100 | b | 100 | c | 200 | b |
| **5b** | 100 | d | 94 | e | 194 | f | 100 | b | 100 | d | 200 | e | 99 | a | 100 | b | 199 | b | 100 | b | 100 | c | 200 | b |
| **average** | 95 |  | 77 |  | 172 |  | 99 |  | 83 |  | 182 |  | 99 |  | 94 |  | 193 |  | 100 |  | 98 |  | 197 |  |
| **1c** | 82 | abc | 52 | abc | 134 | abc | 38 | a | 54 | ab | 92 | a | 94 | a | 92 | ab | 186 | ab | 95 | b | 96 | c | 191 | b |
| **2c** | 65 | a | 58 | abcd | 123 | ab | 91 | b | 56 | abc | 147 | bc | 87 | a | 91 | ab | 177 | ab | 80 | a | 80 | ab | 161 | a |
| **3c** | 88 | bcd | 75 | bcde | 162 | cdef | 92 | b | 60 | abc | 152 | bcd | 85 | a | 88 | a | 172 | a | 98 | b | 92 | c | 191 | b |
| **4c** | 100 | d | 45 | ab | 145 | bcd | 100 | b | 77 | abcd | 177 | bcde | 99 | a | 93 | ab | 192 | ab | 100 | b | 100 | c | 200 | b |
| **5c** | 94 | bcd | 85 | de | 178 | def | 92 | b | 86 | bcd | 178 | bcde | 97 | a | 94 | ab | 191 | ab | 100 | b | 99 | c | 199 | b |
| **average** | 86 |  | 63 |  | 149 |  | 83 |  | 67 |  | 149 |  | 92 |  | 92 |  | 184 |  | 95 |  | 94 |  | 188 |  |
| **1d** | 89 | bcd | 70 | abcde | 159 | bcdef | 32 | a | 47 | a | 80 | a | 100 | a | 86 | a | 186 | ab | 97 | b | 99 | c | 196 | b |
| **2d** | 67 | a | 42 | a | 109 | a | 90 | b | 70 | abcd | 159 | bcd | 99 | a | 95 | ab | 193 | ab | 78 | a | 71 | a | 149 | a |
| **3d** | 87 | bcd | 70 | abcde | 157 | bcde | 93 | b | 91 | cd | 184 | de | 91 | a | 88 | ab | 179 | ab | 98 | b | 96 | c | 194 | b |
| **4d** | 100 | d | 76 | bcde | 176 | def | 92 | b | 67 | abcd | 160 | bcd | 100 | a | 95 | ab | 195 | ab | 100 | b | 100 | c | 200 | b |
| **5d** | 97 | bcd | 86 | de | 183 | ef | 91 | b | 87 | bcd | 178 | cde | 100 | a | 94 | ab | 194 | ab | 99 | b | 99 | c | 199 | b |
| **average** | 88 |  | 69 |  | 157 |  | 80 |  | 73 |  | 152 |  | 98 |  | 91 |  | 189 |  | 94 |  | 93 |  | 188 |  |
| **average**  **total** | 90 |  | 69 |  | 159 |  | 89 |  | 73 |  | 162 |  | 96 |  | 93 |  | 189 |  | 97 |  | 95 |  | 192 |  |
| **LSD_0.05_** | 14 |  | 25 |  | 29 |  | 16 |  | 29 |  | 30 |  | 17 |  | 10 |  | 20 |  | 10 |  | 10 |  | 14 |  |
| **azadirachtin^b^** | 99 |  | 91 |  | 190 |  | 100 |  | 85 |  | 185 |  | 100 |  | 92 |  | 192 |  | 100 |  | 94 |  | 194 |  |

b - (Łozowicka i inn., 2007); B. Łozowicka, P. Kaczyński, J. Nawrot, J. Wysocka .2007. Aktywność deterentna nowych pochodnych alfa-asaronu, Progress in Plant Protection, 47, 303-309
